# Pediatric glioma and medulloblastoma risk and population demographics: a Poisson regression analysis

**DOI:** 10.1093/noajnl/vdaa089

**Published:** 2020-07-22

**Authors:** Ivo S Muskens, Qianxi Feng, Stephen S Francis, Kyle M Walsh, Roberta Mckean-Cowdin, William J Gauderman, Adam J de Smith, Joseph L Wiemels

**Affiliations:** 1 Department of Preventive Medicine, Center for Genetic Epidemiology, Keck School of Medicine, University of Southern California, Los Angeles, California, USA; 2 Department of Neurosurgery, Division of Neuro and Molecular Epidemiology, University of California, San Francisco, California, USA; 3 Department of Neurosurgery, Duke Cancer Institute, and Children’s Health and Discovery Institute, Duke University, Durham, North Carolina, USA; 4 Division of Biostatistics, Department of Preventive Medicine, Keck School of Medicine, University of Southern California, Los Angeles, California, USA

**Keywords:** ethnicity, pediatric brain tumor, risk factors, SEER, SES

## Abstract

**Background:**

The incidence of pediatric brain tumors varies by race and ethnicity, but these relationships may be confounded by socioeconomic status (SES). In this study, the Surveillance, Epidemiology, and End Results Program (SEER) database was evaluated for associations between race/ethnicity and pediatric glioma and medulloblastoma risk with adjustment for SES.

**Methods:**

Pediatric glioma and medulloblastoma cases from the SEER database (years: 2000–2016) were included. Differences in incidence rates by ethnicity, sex, age, and SES-related factors were evaluated by calculation of age-adjusted incidence rates (AAIRs) and annual percent change (APC). SES-related factors (percentage without less than high school graduation, median household income, and percentage foreign-born) were derived from the census at the county-level (year: 2000). Multivariable Poisson regression models with adjustment for selected covariates were constructed to evaluate risk factors.

**Results:**

The highest AAIRs of pediatric glioma were observed among non-Hispanic Whites (AAIR: 2.91 per 100 000, 95%-CI: 2.84–2.99). An increasing incidence of pediatric glioma by calendar time was observed among non-Hispanic Whites and non-Hispanic Blacks (APC: 0.97%, 95%-CI: 0.28–1.68 and APC: 1.59%, 95%-CI: 0.03–3.18, respectively). Hispanic and non-Hispanic Black race/ethnicity was associated with lower risk when compared with non-Hispanic White (incidence rate ratios [IRRs]: 0.66, 95%-CI: 0.63–0.70; and 0.69, 95%-CI: 0.65–0.74, respectively). For medulloblastoma, the highest AAIR was observed for non-Hispanic Whites with a positive APC (1.52%, 95%-CI: 0.15–2.91). Hispanics and non-Hispanic Blacks had statistically significant lower IRRs compared with non-Hispanic Whites (IRRs: 0.83, 95%-CI: 0.73–0.94; and 0.72, 95%-CI: 0.59–0.87, respectively).

**Conclusion:**

Non-Hispanic White race/ethnicity was associated with higher pediatric glioma and medulloblastoma IRRs in models with adjustments for SES.

**Key Points**

- The incidence of pediatric glioma has statistically significantly increased among non-Hispanic Whites and non-Hispanic Blacks.
- Non-Hispanic White race/ethnicity is associated with increased pediatric glioma and medulloblastoma risk.

**Importance of the Study**

The incidence of pediatric brain tumors varies by race and ethnicity but may be confounded by socioeconomic status (SES). Multivariable Poisson regression modeling based on data from Surveillance, Epidemiology, and End Results Program (SEER) allowed for evaluating associations between race/ethnicity and pediatric glioma and medulloblastoma risk with adjustment for SES on a population-based scale. The highest age-adjusted incidence rates (AAIRs) for both glioma and medulloblastoma were observed among non-Hispanic Whites. Increasing incidence of pediatric glioma was observed among non-Hispanic Whites and non-Hispanic Blacks. Only non-Hispanic Whites showed a statistically significant increase in medulloblastoma incidence. Hispanic and non-Hispanic Black race/ethnicity was associated with both lower glioma and medulloblastoma risk when compared with non-Hispanic Whites with adjustment for SES. These findings suggest that a higher risk of pediatric glioma and medulloblastoma observed among non-Hispanic Whites cannot be fully explained by confounding by SES.

Pediatric brain tumors are responsible for the highest number of cancer-related deaths in children in the United States.^1–3^ Both morbidity and mortality much depend on intracranial location and tumor histology.^1^ The prognosis for higher-grade gliomas, in particular, continues to remain poor, and surgery remains the only effective method to prolong survival in most cases.^1^ Clearly, prevention of disease and individually targeted therapies to minimize morbidity remain a priority.

Barriers to prevention and advancement in treatments to improve outcomes for this population include a lack of understanding of brain tumor risk factors and underlying etiologic mechanisms. Ionizing radiation remains the only environmental exposure that has been established as a risk factor.^4,5^ Syndromes associated with cancer predisposition, such as Li–Fraumeni and Neurofibromatosis types 1 and 2 and select birth defects increase the risk of pediatric brain tumors. However, these likely explain less than 10% of all cases.^6–11^ More general birth characteristics have been identified as risk factors, for example, older maternal and paternal age and increased birth weight, but evidence for these is generally less strong and often inconsistent.^12–15^ Other associations that have less consistently been observed in epidemiologic studies include parental smoking, exposure of parents or children to pesticides, higher socioeconomic status (SES), dietary nitrites or nitrosamines during pregnancy, and parental occupation.^16–19^ Our current understanding of risk factors for pediatric brain tumors is limited and suspected risk factors likely explain the occurrence of brain tumors in a minority of patients.^15,16,20^

Several biologic risk factors for *adult* glioma have been identified, such as select common genetic variants, increased leukocyte telomere length, ancestry (mainly European), and a lack of a history of atopy.^20^ These risk factors may also apply to pediatric glioma, but evidence remains limited.^15,21^ For instance, similar to adults, glioma incidence is highest among non-Hispanic White children in the United States. However, this may be confounded by SES and access to healthcare.^22^ In this study, the Surveillance, Epidemiology, and End Results Program (SEER) database was evaluated for associations between SES-related risk factors and pediatric glioma and medulloblastoma risk using Poisson regression modeling to improve the understanding of the contribution of ethnicity and SES to pediatric glioma and medulloblastoma risk.

## Methods

### Case Ascertainment

The SEER (SEER18, submission date: November 2018) database was used to evaluate patterns in glioma and medulloblastoma during the years 2000–2016. Children aged 0–19 years at diagnosis with glioma and medulloblastoma were included in the study. The following ICD-O-3 codes were used to define glioma cases: 9382, 9383, 9391, 9392, 9393, 9394, 9380, 9381, 9384, 9400, 9410, 9411, 9420, 9401, 9421, 9424, 9425, 9432, 9440, 9441, 9442, 9450, 9451, and 9460 as used by the Central Brain Tumor Registry of the United States (CBTRUS).^22^ A subgroup of glioma cases was created for pilocytic astrocytoma (ICD-O-3: 9421 and 9425). Another subgroup of glioma cases was analyzed separately as cases from California. Cases from California included a large number of Hispanic children that may be more homogenous due to origination from a more similar geographical location and registry site when compared with Hispanics from other regions of the United States covered by SEER.

Medulloblastoma was chosen as a separate group of pediatric brain tumors for comparison. The following ICD-O-3 codes were used to ascertain medulloblastoma cases: 9470, 9471, 9472, and 9474.^22^

### Exposure Assessment

The following racial/ethnic groups were included in the analyses from SEER based on medical records: Hispanic, non-Hispanic Black, non-Hispanic White, Asian/Pacific Islander (API), and American Indian/Alaska Native (AIAN). Hispanics were also identified by SEER using an algorithm that evaluates last names. Age groups were categorized as 0–4, 5–9, 10–14, and 15–19 years. SES was measured using 2 county-level variables based on 2000 US census data: median household income and percentage of people with less than high school education. Additionally, the variable “percentage foreign-born” was obtained from the 2000 US census data for Hispanics. These SES-related variables were categorized by quintiles among all available pediatric cancer cases in SEER (ie, extending beyond the pediatric brain tumor cases included in these analyses).

### Statistical Analysis

Age-adjusted incidence rates (AAIRs) were calculated by sex, age group, median household income in the year 2000 (in quintiles), percentage of people with less than high school education in 2000 (in quintiles), and percentage foreign-born in 2000 (by quintiles) using SEER*Stat v8.3.6. We used R (version 3.6.0) to visualize the calculated AAIRs by ethnicity and by age group over the period 2000–2016. Annual percent change (APC) was calculated by the JoinPoint regression program 4.7.0.0,^23^ with the year of diagnosis as the primary predictor variable. APC was evaluated by the same variables as above for AAIRs. APCs for all variables were also calculated separately for all races/ethnicities.

Poisson regression modeling was utilized to compare AAIRs in a multivariable manner. In these models, the AAIR was the dependent variable and can be denoted as

$$\begin{array}{ll} Count=\; & ethnicity+age+gender+median\;household\;income\\ & +percentage\;without\;high\;school\;graduation\\ & +of\;fset(ln(weighted\;population)), \end{array}$$

where

$$\begin{array}{l} Weighted\;population=\frac{population\;in\;each\;category}{\left(\frac{standard\;population\;in\;each\;category}{total\;standard\;population}\right)} \end{array}$$

The 2000 U.S. Standard Population was used for age-standardization.^24^ Variables entered into the model were sex, age group, race/ethnicity, median household income in 2000, and percentage of people with less than high school education in 2000. Non-Hispanic White race/ethnicity and female sex were used as reference categories. For variables categorized into quintiles, we used the lowest quintile as the reference. Incidence rate ratios (IRRs) were calculated for each category. We also evaluated the differences in change in the trend of AAIR by race/ethnicity using a model that included race/ethnicity, year of diagnosis, and an interaction term for race/ethnicity and year of diagnosis. Generalized variance inflation factors (GVIFs) were used to determine multicollinearity. Any variable with a GVIF^(1/(2×degree of freedom))^ greater than 2 was deemed to be problematic. Multivariable models were visualized using forest plots in R using the “forestplot” package.^25^

For glioma, multivariable models were constructed for all racial/ethnic combinations of non-Hispanic White, non-Hispanic Black, Hispanic, and API. It was deemed that the sample size for AIAN did not allow for multivariable modeling (*N* = 66). For medulloblastoma, the same multivariable models were constructed with the exception of the exclusion of children in the API category due to the small sample size (*N* = 115). Additional models that also included percentage of foreign-borns were created for Hispanics due to more recent migration into the United States at a population level. Percentage foreign-born was not included in multivariable models for other racial/ethnic groups (apart from Hispanics) due to small sample size and hence, an inability to create balanced quintiles. *P*-values for trend were calculated by entering ordinal variables as numeric into the models. *P*-values smaller than .05 were interpreted to be statistically significant.

## Results

### Glioma

**Figure 1.**
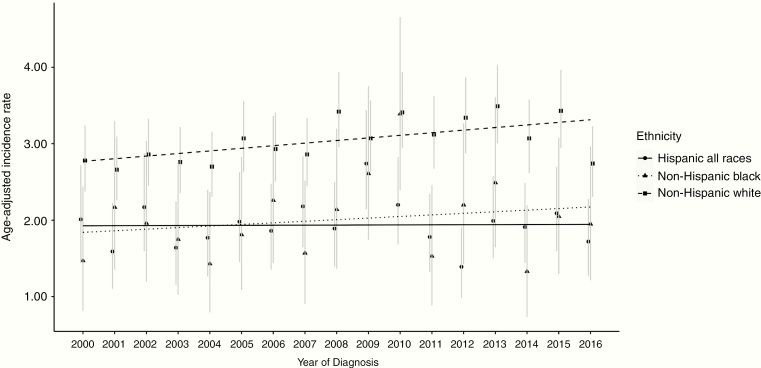
Age-adjusted incidence rates (AAIRs) for glioma by ethnicity from 2000 to 2016. The AAIRs and 95%-CIs for glioma were derived from SEER years 2000 to 2016. Each line depicts a different ethnicity. Non-Hispanic Black and non-Hispanic White showed a statistically significant positive annual percent change (APC) over the period 2000–2016 (non-Hispanic Black APC: 1.59, 95%-CI: 0.03–3.18, non-Hispanic White APC: 0.97, 95%-CI: 0.28–1.68). SEER, Surveillance, Epidemiology, and End Results Program.

A total of 9449 glioma cases were ascertained, of which the majority were non-Hispanic White (*N* = 5490). The overall AAIR for pediatric glioma was 2.37 per 100 000 (95%-CI: 2.32–2.43), with non-Hispanic Whites having the highest incidence rate (AAIR 2.91 per 100 000 people per year, 95%-CI: 2.84–2.99). There was a statistically significant overall positive APC over the period from 2000 to 2016 (0.59%, 95%-CI: 0.08–1.10). When rates were evaluated separately by race/ethnicity, a statistically significant positive APC was identified among children who were non-Hispanic White and non-Hispanic Black (APCs: 0.97%, 95%-CI: 0.28–1.68 and 1.59%, 95%-CI: 0.03–3.18, respectively), but not among children who were Hispanics or API (APCs: −0.22%, 95%-CI: −1.26-0.84 and 0.12%, 95%-CI: −1.79–2.06, respectively, Figure 1 and Supplementary Table S1). In the overall analysis, APC was also statistically significant among the 10–14-year age group (APC: 0.65%, 95%-CI: 0.03–1.27); positive trends that did not reach statistical significance were observed in other age groups. The interaction model showed that the slopes for change in AAIR trends from 2000 to 2016 were marginally different when comparing change for children who are Hispanic compared with non-Hispanic Whites or non-Hispanic Blacks (likelihood-ratio χ ^2^(2) = 7.57; *P* = .02, Supplementary Table S2).

**Figure 2.**
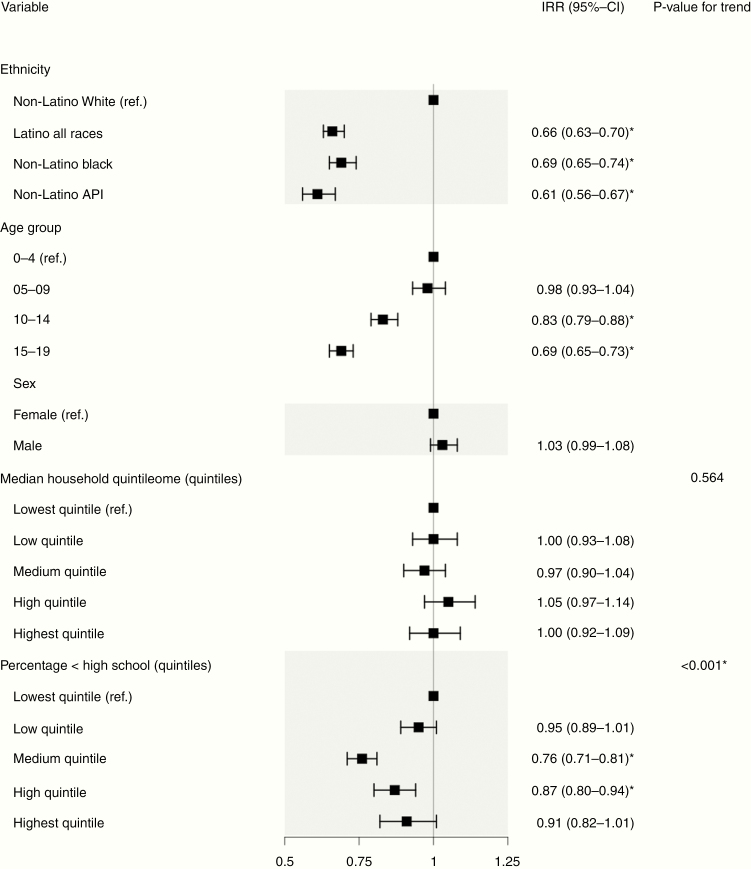
Multivariable Poisson regression model of age-adjusted incidence rates (AAIRs) in glioma. This forest plot depicts incidence rate ratios (IRRs) for various risk factors and association with glioma. IRRs were derived from a Poisson regression model using AAIRs as the dependent variable. A total of 9265 subjects with complete information were included in the model. *Statistically significant IRR.

**Figure 3.**
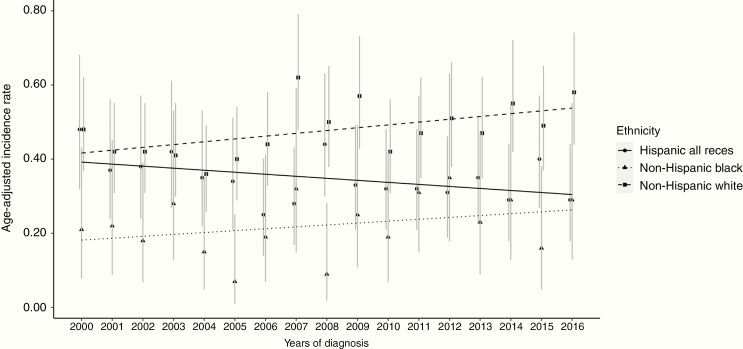
Age-adjusted incidence rates (AAIRs) for medulloblastoma by ethnicity from 2000 to 2016. The AAIRs per 100 000 persons and 95%-CIs for medulloblastoma were derived from SEER years 2000 to 2016. Each line depicts a different ethnicity. Non-Hispanic White showed a statistically significant positive annual percent change (APC) over the period 2000–2016 (APC: 1.52, 95%-CI: 0.15–2.91). SEER, Surveillance, Epidemiology, and End Results Program.

In the overall multivariable Poisson regression model, children who were Hispanic, non-Hispanic Black, and non-Hispanic APIs had a statistically significant lower risk of glioma compared with non-Hispanic Whites (IRRs: 0.66, 95%-CI: 0.63–0.70, 0.69, 95%-CI: 0.65–0.74, and 0.61, 95%-CI: 0.56–0.67, respectively, Figure 2). Older age group was associated with lower IRR (IRR age group 10–14 years: 0.83, 95%-CI: 0.79–0.88 and IRR age group 15–19 years: 0.69, 95%-CI: 0.65–0.73 with age group 0–4 years as reference, Figure 3). A higher county-level percentage of people with less than high school education in 2000 was associated with decreased glioma risk (IRR medium quintile: 0.76, 95%-CI: 0.71–0.81 and IRR high quintile: 0.87, 95%-CI: 0.80–0.94 with the lowest quintile as reference, *P*-value for trend <.001, Figure 2). Male sex was not associated with increased IRR compared with female (IRR: 1.03, 95%-CI: 0.99–1.08). Multivariable models for children who were non-Hispanic White, non-Hispanic Black, and Hispanic showed similar associations (Supplementary Figure S1a, S1b, and S1c). The addition of county-level percentage of foreign-borns in 2000 to the model for Hispanic children showed that a higher percentage of foreign-borns was associated with decreased risk (*P*-value for trend = .005, Supplementary Figure S1d). With the models adjusted for only ethnicity, gender, and age, we found that the IRRs of those variables did not change greater than 20% for glioma compared with the full model. A statistically significant interaction was detected between age group and county-level median household income [likelihood χ ^2^(12) = 22.06; *P* = .037], but not between age group and county-level percentage of people with less than high school education percentage or ethnicity. No substantial evidence of collinearity was identified for the regression models (all adjusted GVIF <10).

A total of 3267 pilocytic astrocytoma cases were ascertained from SEER. Generally, all models showed associations that were very similar to the associations identified in the overall glioma models (Supplementary Table S3). An analysis that only included glioma cases from California (*N* = 3644) also showed that AAIR was highest among children who were non-Hispanic White and over time there was a positive APC (non-Hispanic Whites: AAIR 2.75, 95%-CI: 2.61–2.89, APC: 1.29, 95%-CI: 0.36–2.23, Supplementary Table S4).

### Medulloblastoma

A total of 1552 medulloblastoma cases were identified in SEER with an overall AAIR of 0.39 per 100 000 (95%-CI: 0.37–0.41). The largest number of children impacted by medulloblastoma was non-Hispanic White (*N* = 887), who correspondingly had the highest AAIR (AAIR: 0.48 per 100 000, 95%-CI: 0.44–0.51). The lowest AAIR (0.22 per 100 000, 95%-CI: 0.18–0.27) was observed among children who were non-Hispanic Black. There was a positive overall APC for all children combined from 2000 to 2016, but the CIs included the null (0.86%, 95%-CI: −0.10 to 1.84). When APC was evaluated stratified by race/ethnicity, a positive trend was observed for children who were non-Hispanic White (APC: 1.52%, 95%-CI: 0.15–2.91) that was statistically significant; positive APCs also were observed for children who were non-Hispanic Black, or API (APC: 2.01%, 95%-CI: −1.50 to 5.63, and 3.66%, 95%-CI: −0.47 to 7.97, respectively, Figure 3 and Supplementary Table S5) but the CIs included the null. A negative APC that was consistent with the null was observed for children who were Hispanic (APC: −1.46%, 95%-CI: −3.08 to 0.19).

**Figure 4.**
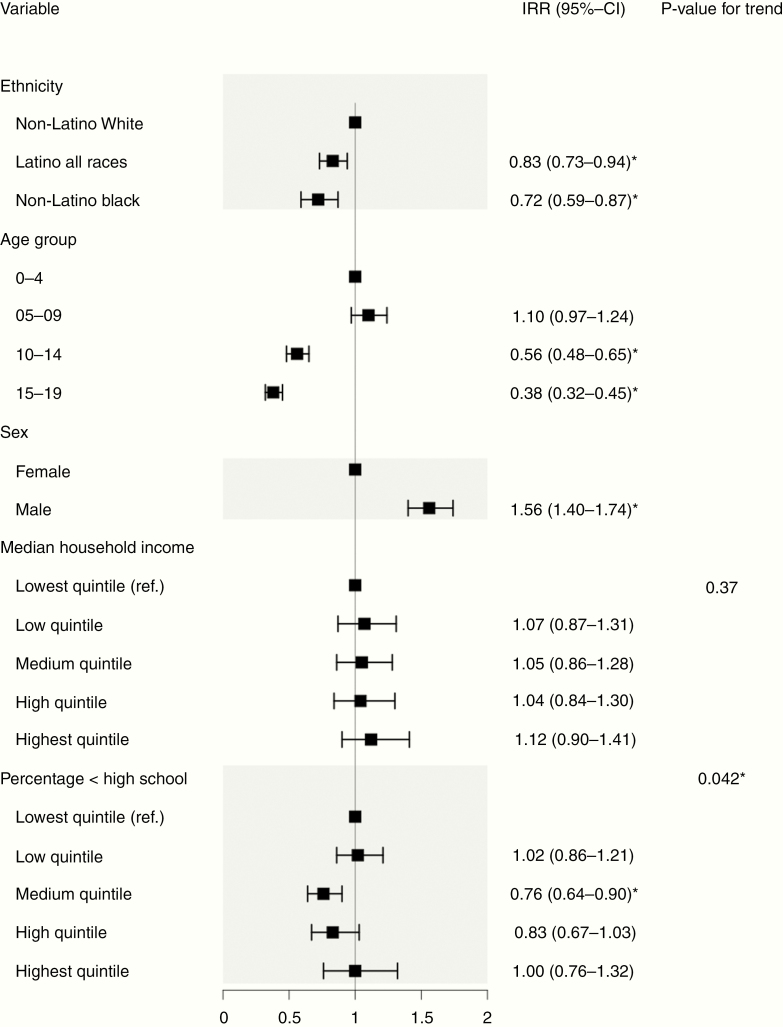
Multivariable Poisson regression model of age-adjusted incidence rates (AAIRs) in medulloblastoma. This forest plot depicts incidence rate ratios (IRRs) for various risk factors and association with medulloblastoma. IRRs were derived from a Poisson regression model using AAIRs as the dependent variable. A total of 1417 subjects with complete information were included in the model. *Statistically significant IRR.

Children who were Hispanic and non-Hispanic Black had statistically significant lower IRRs compared with children who were non-Hispanic White (IRR: 0.83, 95%-CI: 0.73–0.94 and 0.72, 95%-CI: 0.59–0.87, respectively, Figure 4). Similar to glioma, the older age group was associated with lower IRR (IRR in age group 10–14 years: 0.56, 95%-CI: 0.48–0.65 and 0.38, 95%-CI: 0.32–0.45, respectively, with age group 0–4 years as reference). Male sex showed a statistically significant association with increased IRR compared with female (IRR: 1.56, 95%-CI: 1.40–1.74). A higher percentage of county-level people with less than high school education showed a borderline statistically significant association with decreased risk (*P*-value for trend = .042). Similar associations were identified in multivariable models stratified by race/ethnicity (Supplementary Figure S2a, S2b, and S2c). A county-level higher percentage of foreign-borns was associated with a decreased risk in Hispanics when county-level percentage foreign-born was added as a variable to the model (*P*-value for trend <.001, Supplementary Figure S2d). With the models adjusted for only ethnicity, gender, and age, we found that the IRR of non-Hispanic Black decreased by 34.72% [IRR = 0.72 versus IRR = 0.47], suggesting the necessity to adjust for the SES variables (data not shown).

No statistically significant interactions were identified between age group, median household income, high school education, or ethnicity. No substantial evidence of collinearity was identified for the regression models (all adjusted GVIF <10).

## Discussion

This study aimed to evaluate trends in the incidence of pediatric glioma and medulloblastoma based on high-quality SEER data. Poisson regression modeling allowed for us to assess race/ethnicity and SES-related risk factors for association with pediatric glioma and medulloblastoma in finer detail than prior population-based studies and among a large number of children with the adjustment of covariates. We observed a low but statistically significant increasing rate of pediatric glioma among non-Hispanic White (APC: 0.97%) and non-Hispanic Black children (APC: 1.59%) over a 16-year period from 2000 to 2016, whereas a statistically significant APC for medulloblastoma was only identified for non-Hispanic Whites (APC: 1.52%).

In multivariable modeling, children who were non-Hispanic White were at higher risk of both glioma and medulloblastoma than children from other racial/ethnic backgrounds. Clear patterns by SES indicators did not explain the association between race/ethnicity and pediatric brain tumors as the association persisted after controlling for education and income. A lower percentage of people with less than high school education at the county-level was associated with a higher risk in the multivariable model, but the median household income was not. This finding may perhaps be explained by the residual confounding of access to healthcare or healthcare insurance. Patterns were similar when statistical models were restricted to pediatric pilocytic astrocytoma. When we restricted our analysis to children from California, the AAIR also was highest among children who were non-Hispanic White. This suggests that the higher AAIR observed among non-Hispanic Whites in all SEER cases is unlikely to be the result of confounding due to sociodemographic or geographical differences between the large number of non-Hispanic White or Hispanic children in California and children from other racial/ethnic groups in other state registries.

Studies that utilized the SEER and National Program of Cancer Registries (NPCR) databases have identified differences in incidence rates in pediatric brain tumors over time.^26,27^ One study that evaluated both SEER and NPCR also found a statistically significant increase in pediatric glioma and pilocytic astrocytoma incidence but not in medulloblastoma incidence between 1998 and 2013.^26^ An analysis by ethnicity showed that non-Hispanic Whites had a statistically significant positive APC over the same period for glioma.^26^ The authors suggested that the increase in incidence may be the result of improved accessibility and higher sensitivity of detection methods such as MRI as well as increased surveillance of patients at risk of brain tumors.^26^ Another study that evaluated SEER between 1973 and 2009 showed a statistically significant increase of the incidence of pathology not otherwise specified had between 1996 and 2009, whereas pilocytic astrocytoma did not.^27^

To our knowledge, the association between race/ethnicity and pediatric glioma and medulloblastoma with this level of detail and adjustment for SES-related factors has not been reported before. Studies evaluating pediatric brain tumor incidence rates between specific countries show varying incidence rates, which may reflect a similar association but may also be the result of local differences in etiologic exposures and SES.^16,22,28,29^ Most of these studies were conducted among predominantly non-Hispanic White populations and may, therefore, provide less insight into ethnicity-related risk.^13,28,30,31^ Non-Hispanic White race/ethnicity has been associated with higher glioma incidence among adults, with 2 genomic regions related to European ancestry associated with increased adult glioma risk based on local admixture estimation.^32,33^ This may also explain the observed association between higher percent of foreign-borns and lower glioma and medulloblastoma risk in Hispanics, as Hispanics that migrated more recently tend to have lower European ancestry based on global admixture estimation.^34^ Therefore, common germline variants and haplotypes that are associated with increased adult glioma risk, of which some are related to European ancestry, may also be associated with increased pediatric glioma risk.^35^ Indeed, some of the same single nucleotide polymorphisms associated with increased adult glioma risk have recently been associated with glioma risk among adolescents and young adults up to 29 years of age.^21^ Nevertheless, the observed difference in pediatric brain glioma incidence by ethnicity with adjustment for SES-related factor warrants further investigation of common genetic variation for association with glioma risk.

This study has various strengths. The size of the SEER program allowed for a high number of pediatric glioma and medulloblastoma cases from several racial/ethnic backgrounds to be ascertained and APCs and AAIR to be calculated and evaluated over a 16-year period. Population-based studies have reported differences in incidence rates by race/ethnicity, potentially due to selection bias or over the participation of higher SES groups.^1^ In SEER data, we see that race/ethnicity is an important predictor of pediatric brain tumor rates even when accounting for SES in the same model.^27^ In addition, selection bias is minimized since we have included all available cases in the cancer registry, which has mandatory reporting. To our knowledge, this is the first study that used Poisson regression to allow for multivariable modeling of both SES and ethnicity risk factors to simultaneously control for the contributions of both factors to pediatric brain tumor risk.

This study is also limited in various aspects. We did not include cases from before 2000 as SEER 18 only includes cases from 2000 onwards. Also, the introduction of the MRI in the 1980s and subsequent improvement of brain tumor detection may have caused an uptick in pediatric brain tumor patients included in SEER before 2000,^27^ but less likely so during our observation period. It has also been suggested that children with low SES still have relatively limited access to advanced imaging due to varying insurance status, which may have resulted in participation bias.^27^ The SEER18 registry only covers 27.8% of the total US population and the SES-related variables used in this study are community-level measurements based on census data from 2000.^36^ The inclusion of those covariates may lead to residual confounding since the community-level measurements cannot fully represent the measurements at the level of the individual. In our models, only reported ethnicity was evaluated which limits the genetic implications from our models, as reported ethnicity and corresponding genetic ancestry may be different.^37–39^ Poisson regression models were constructed as matched controls were not available through SEER, and quintiles were derived from all available pediatric cancer cases in SEER, which may further limit the extrapolatability of our findings to the general population. Therefore, our results warrant replication in other multiethnic cohorts with individual-level data. We also restricted our analyses to the main subgroups of pediatric glioma and did not perform subgroup analyses in medulloblastoma due to small remaining sample sizes and recent changes in subgroup stratification, thus not permitting evaluation of historic data.

In conclusion, this study provides evidence that race/ethnicity is an important predictor of pediatric glioma and medulloblastoma risk even after adjustment for measures of SES in multivariable models. This insight may provide new directions for future research aimed at discovering European ancestry-associated haplotypes or rare genetic variants that confer pediatric glioma or medulloblastoma risk, or evaluation of gene–environment interactions. Continued changes in annual rates that are not fully attributable to diagnostic methodology suggest that environmental factors that vary over time are worthy of study as well to identify *modifiable* risk factors for pediatric brain cancers. However, our findings do need to be replicated, preferably in another nationally representative database to ensure reproducibility of the results, or in a multiethnic case–control fashion with adequate adjustment for SES to increase the validity of the study.

## Supplementary Material

vdaa089_suppl_Supplementary_Material

## Notes

**Authorship Statement**. Analysis: IM and QF. Interpretation of results: IM, QF, SF, KW, RMC, AS, and JW. Manuscript preparation: IM and QF. Analysis supervision: JG and JW. Review of the manuscript: SF, KW, RM, JW, AS, and JG.
