# Supplementary material for: Pediatric glioma and medulloblastoma risk and population demographics: a Poisson regression analysis
Source: Neurooncol Adv. 2020 Jul 22;2(1):vdaa089. doi: 10.1093/noajnl/vdaa089 (PMC7447139; doi:10.1093/noajnl/vdaa089)
Supplement: vdaa089_suppl_Supplementary_Material [file vdaa089_suppl_supplementary_material.docx]

Supplementary tables and figures

Supplementary table 1: Age-Adjusted Incidence Rates (AAIR; SEER18, 2000-2016) for gliomas by selected demographic characteristics

|  | **Overall** | | | **Non-Hispanic White** | | | **Hispanic all races** | | |
| --- | --- | --- | --- | --- | --- | --- | --- | --- | --- |
|  | **N** | **AAIR^1^**  **(95%-CI)** | **APC^2^**  **(95%-CI)** | **N** | **AAIR^1^**  **(95%-CI)** | **APC^2^**  **(95%-CI)** | **N** | **AAIR^1^**  **(95%-CI)** | **APC^2^**  **(95%-CI)** |
| **Overall** | 9,449 | 2.37  (2.32, 2.42) | **0.59***  **(0.08, 1.1)** | 5,490 | 2.91  (2.84, 2.99) | **0.97***  **(0.28, 1.68)** | 2,145 | 1.82  (1.75, 1.90) | -0.22  (-1.26, 0.84) |
| **Gender** |  |  |  |  |  |  |  |  |  |
| ***Male*** | 4,920 | 2.41  (2.34, 2.48) | 0.55  (-0.06, 1.16) | 2,882 | 2.98  (2.87, 3.09) | 0.71  (-0.06, 1.48) | 1,092 | 1.82  (1.71, 1.93) | -0.07  (-1.68, 1.57) |
| ***Female*** | 4,529 | 2.33  (2.26, 2.40) | 0.60  (-0.12, 1.33) | 2,608 | 2.85  (2.74, 2.96) | **1.27***  **(0.26, 2.30)** | 1,053 | 1.83  (1.72, 1.94) | -0.42  (-1.43, 0.60) |
| **Age (years)** |  |  |  |  |  |  |  |  |  |
| ***0-4*** | 2,738 | 2.82  (2.71, 2.93) | 0.22  (-0.51, 0.96) | 1,516 | 3.42  (3.25, 3.60) | 0.74  (-0.23, 1.73) | 694 | 2.26  (2.09, 2.43) | -0.86  (-2.26, 0.56) |
| ***5-9*** | 2,588 | 2.64  (2.54, 2.75) | 0.47  (-0.49, 1.43) | 1,444 | 3.15  (2.99, 3.31) | 0.90  (-0.46, 2.27) | 645 | 2.20  (2.03, 2.38) | -0.88  (-2.76, 1.03) |
| ***10-14*** | 2,233 | 2.20  (2.11, 2.29) | **0.65***  **(0.03, 1.27)** | 1,359 | 2.77  (2.62, 2.92) | 0.59  (-0.13, 1.32) | 445 | 1.55  (1.41, 1.70) | 0.81  (-1.50, 3.18) |
| ***15-19*** | 1,890 | 1.85  (1.77, 1.94) | 1.28  (-0.03, 2.60) | 1,171 | 2.33  (2.20, 2.47) | **1.72***  **(0.16, 3.30)** | 361 | 1.30  (1.17, 1.44) | 0.35  (-1.88, 2.63) |
| **Median household income in 2000^3^** |  |  |  |  |  |  |  |  |  |
| ***Lowest*** | 1,930 | 2.33  (2.23, 2.44) | 0.62  (-0.34, 1.59) | 1,215 | 2.77  (2.62, 2.93) | **1.29***  **(0.14, 2.45)** | 351 | 1.79  (1.61, 1.99) | -0.68  (-2.81, 1.50) |
| ***Low*** | 1,949 | 2.08  (1.99, 2.18) | 0.43  (-0.40, 1.27) | 859 | 2.76  (2.58, 2.95) | 0.26  (-0.85, 1.37) | 711 | 1.70  (1.57, 1.83) | 0.07  (-1.78, 1.95) |
| ***Medium*** | 1,786 | 2.47  (2.35, 2.58) | 0.88  (-0.17, 1.94) | 1,104 | 3.04  (2.86, 3.22) | **1.99***  **(0.81, 3.18)** | 406 | 1.93  (1.75, 2.13) | -1.82  (-4.22, 0.63) |
| ***High*** | 1,882 | 2.61  (2.49, 2.73) | 0.78  (-0.46, 2.02) | 1,163 | 3.09  (2.91, 3.27) | 0.73  (-0.46, 1.93) | 299 | 2.04  (1.81, 2.28) | 1.28  (-1.42, 4.05) |
| ***Highest*** | 1,899 | 2.46  (2.35, 2.58) | 0.12  (-0.91, 1.16) | 1,149 | 2.91  (2.75, 3.09) | 0.51  (-0.83, 1.87) | 377 | 1.85  (1.67, 2.05) | 0.22  (-2.06, 2.56) |
| **Percentage < high school education in 2000^4^** |  |  |  |  |  |  |  |  |  |
| ***Lowest*** | 2,010 | 2.84  (2.72, 2.97) | 0.17  (-1.22, 1.57) | 1,473 | 3.08  (2.92, 3.24) | 0.16  (-1.05, 1.39) | 216 | 2.19  (1.90, 2.50) | -0.87  (-3.94, 2.30) |
| ***Low*** | 1,790 | 2.39  (2.28, 2.50) | 0.35  (-0.31, 1.01) | 1,094 | 2.97  (2.79, 3.15) | **1.03***  **(0.25, 1.82)** | 282 | 1.77  (1.57, 1.99) | -0.44  (-2.89, 2.06) |
| ***Medium*** | 2,013 | 2.46  (2.35, 2.56) | **1.33***  **(0.18, 2.49)** | 1,178 | 2.88  (2.72, 3.05) | **2.11***  **(0.75, 3.49)** | 447 | 1.95  (1.77, 2.13) | 0.41  (-2.00, 2.88) |
| ***High*** | 1,770 | 2.23  (2.12, 2.33) | **1.18***  **(0.07, 2.30)** | 940 | 2.82  (2.64, 3.01) | **1.43***  **(0.18, 2.70)** | 439 | 1.83  (1.66, 2.01) | -0.16  (-2.90, 2.65) |
| ***Highest*** | 1,863 | 2.05  (1.96, 2.14) | 2000-2003: 11.01  (-2.61, 26.52) | 805 | 2.74  (2.55, 2.93) | 0.16  (-1.12, 1.45) | 760 | 1.70  (1.58, 1.82) | -0.46  (-2.41, 1.53) |
|  |  |  | **2003-2016:**  **-1.53***  **(-2.80, -0.24)** |  |  |  |  |  |  |
| **Percentage foreign-born in 2000^5^** |  |  |  |  |  |  |  |  |  |
| ***Lowest*** | 1,913 | 2.64  (2.52, 2.76) | **1.37***  **(0.08, 2.67)** | 1,460 | 2.88  (2.74, 3.04) | **1.62***  **(0.37, 2.89)** | 88 | 2.01  (1.61, 2.48) | -0.55  (-5.55, 4.72) |
| ***Low*** | 1,941 | 2.64  (2.53, 2.76) | 0.63  (-0.22, 1.48) | 1,379 | 3.00  (2.84, 3.16) | 0.86  (-0.13, 1.87) | 227 | 2.00  (1.75, 2.28) | **-2.48***  **(-4.82, -0.08)** |
| ***Medium*** | 1,867 | 2.57  (2.46, 2.69) | 0.29  (-1.02, 1.63) | 1,182 | 3.08  (2.90, 3.26) | 0.35  (-1.10, 1.83) | 266 | 1.85  (1.63, 2.09) | 0.22  (-2.70, 3.24) |
| ***High*** | 1,853 | 2.12  (2.03, 2.22) | 0.21  (-0.59, 1.03) | 785 | 2.75  (2.56, 2.95) | **1.47***  **(0.24, 2.72)** | 768 | 1.86  (1.73, 2.00) | -0.74  (-2.12, 0.66) |
| ***Highest*** | 1,872 | 2.03  (1.94, 2.12) | 0.27  (-1.10, 1.66) | 684 | 2.76  (2.55, 2.97) | 0.29  (-1.44, 2.06) | 795 | 1.72  (1.60, 1.84) | 0.53  (-1.70, 2.82) |
|  | **Non-Hispanic Black** | | | **Non-Hispanic API** | | | **Non-Hispanic AIAN** | | |
|  | **N** | **AAIR^1^**  **(95%-CI)** | **APC^2^**  **(95%-CI)** | **N** | **AAIR^1^**  **(95%-CI)** | **APC^2^**  **(95%-CI)** | **N** | **AAIR^1^**  **(95%-CI)** | **APC^2^**  **(95%-CI)** |
| **Overall** | 1,046 | 1.93  (1.81, 2.05) | **1.59***  **(0.03, 3.18)** | 576 | 1.68  (1.55, 1.82) | 0.12  (-1.79, 2.06) | 66 | 1.65  (1.27, 2.10) | NA |
| **Gender** |  |  |  |  |  |  |  |  |  |
| ***Male*** | 533 | 1.93  (1.77, 2.10) | 1.67  (-0.48, 3.86) | 315 | 1.79  (1.60, 2.00) | 1.07  (-1.26, 3.46) | 35 | 1.75  (1.22, 2.44) | NA |
| ***Female*** | 513 | 1.92  (1.76, 2.10) | 1.57  (-0.43, 3.61) | 261 | 1.56  (1.38, 1.76) | -0.81  (-3.65, 2.11) | 31 | 1.54  (1.05, 2.19) | NA |
| **Age (years)** |  |  |  |  |  |  |  |  |  |
| ***0-4*** | 298 | 2.32  (2.06, 2.60) | 0.81  (-1.83, 3.51) | 168 | 2.00  (1.71, 2.33) | 1.13  (-1.86, 4.21) | 16 | 1.72  (0.98, 2.80) | NA |
| ***5-9*** | 299 | 2.24  (2.00, 2.51) | 1.80  (-0.83, 4.51) | 154 | 1.83  (1.55, 2.14) | -0.94  (-4.02, 2.24) | 15 | 1.58  (0.88, 2.60) | NA |
| ***10-14*** | 255 | 1.80  (1.59, 2.04) | 1.61  (-0.34, 3.60) | 133 | 1.54  (1.29, 1.83) | 1.88  (-2.14, 6.06) | 17 | 1.63  (0.95, 2.61) | NA |
| ***15-19*** | 194 | 1.36  (1.17, 1.56) | 2.10  (-0.91, 5.19) | 121 | 1.36  (1.13, 1.63) | -0.06  (-4.50, 4.59) | 18 | 1.67  (0.99, 2.64) | NA |
| **Median household income in 2000^3^** |  |  |  |  |  |  |  |  |  |
| ***Lowest*** | 294 | 1.86  (1.65, 2.08) | 0.31  (-1.88, 2.55) | 36 | 1.82  (1.28, 2.53) | NA | 16 | 0.92  (0.53, 1.50) | NA |
| ***Low*** | 246 | 1.95  (1.72, 2.21) | **3.74***  **(0.70, 6.88)** | 115 | 1.48  (1.22, 1.78) | -0.43  (-4.90, 4.25) | 4 | 1.10  (0.30, 2.83) | NA |
| ***Medium*** | 179 | 1.83  (1.58, 2.12) | 1.98  (-1.99, 6.12) | 74 | 1.54  (1.21, 1.93) | NA | 7 | 1.19  (0.48, 2.47) | NA |
| ***High*** | 183 | 2.00  (1.72, 2.31) | 0.87  (-2.24, 4.07) | 167 | 1.74  (1.49, 2.03) | -0.53  (-4.02, 3.08) | 32 | 2.86  (1.95, 4.03) | NA |
| ***Highest*** | 144 | 2.07  (1.75, 2.44) | 0.40  (-4.10, 5.11) | 183 | 1.79  (1.54, 2.07) | 0.42  (-2.58, 3.51) | 6 | 2.67  (0.98, 5.87) | NA |
| **% < High school education in 2000^4^** |  |  |  |  |  |  |  |  |  |
| ***Lowest*** | 123 | 1.90  (1.58, 2.27) | 1.32  (-3.54, 6.41) | 122 | 2.27  (1.88, 2.71) | -0.99  (-6.41, 4.74) | 39 | 3.13  (2.22, 4.27) | NA |
| ***Low*** | 205 | 2.02  (1.75, 2.32) | -1.05  (-3.83, 1.81) | 170 | 1.50  (1.28, 1.75) | 1.82  (-1.09, 4.81) | 11 | 1.58  (0.79, 2.83) | NA |
| ***Medium*** | 223 | 2.28  (1.99, 2.61) | 2.22  (-0.60, 5.12) | 127 | 1.60  (1.33, 1.90) | -0.75  (-3.64, 2.22) | 4 | 0.90  (0.24, 2.31) | NA |
| ***High*** | 316 | 1.75  (1.56, 1.95) | **3.45***  **(0.31, 6.68)** | 52 | 1.57  (1.17, 2.06) | 1.92  (-4.44, 8.71) | 10 | 1.20  (0.58, 2.22) | NA |
| ***Highest*** | 179 | 1.82  (1.56, 2.11) | -0.40  (-3.93, 3.27) | 104 | 1.64  (1.34, 1.99) | -0.77  (-5.63, 4.35) | 1 | 0.11  (0.00, 0.67) | NA |
| **Percentage foreign-born in 2000^5^** |  |  |  |  |  |  |  |  |  |
| ***Lowest*** | 316 | 2.03  (1.81, 2.27) | 2000-2013:  3.4  (-0.06, 6.98) | 20 | 2.13  (1.30, 3.29) | NA | 7 | 0.58  (0.23, 1.20) | NA |
|  |  |  | 2013-2016:  -16.98  (-40.66, 16.15) |  |  |  |  |  |  |
| ***Low*** | 235 | 1.95  (1.71, 2.22) | 1.17  (-1.27, 3.67) | 36 | 1.40  (0.98, 1.94) | NA | 36 | 2.24  (1.57, 3.10) | NA |
| ***Medium*** | 241 | 1.95  (1.71, 2.21) | 2.20  (-1.02, 5.54) | 126 | 1.79  (1.49, 2.13) | NA | 17 | 3.59  (2.09, 5.74) | NA |
| ***High*** | 113 | 1.52  (1.25, 1.82) | 1.74  (-2.04, 5.66) | 162 | 1.69  (1.44, 1.97) | 0.16  (-2.84, 3.26) | 3 | 0.55  (0.11, 1.70) | NA |
| ***Highest*** | 141 | 2.05  (1.72, 2.41) | 1.87  (-2.61, 6.56) | 231 | 1.64  (1.43, 1.86) | 0.37  (-2.03, 2.84) | 2 | 0.74  (0.09, 2.74) | NA |

Supplementary table 1 legend:

^1^ AAIR per 100,000 persons, diagnosed from 2000-2016, SEER18 data.

^2^ Annual percent change (APC) for 2000-2016 using SEER18 data.

^3^ Median household income by county in 2000 from the Census 2000 ACS data. Lowest: less than $39,010; low: $39,011-$42,190; medium: $42,191-$47,320; high: $47,321-$57,310; highest: more than $57,311.

^4^ Percent of people less than high school education by county in 2000 from the Census 2000 ACS data. Lowest: less than 13.41%; low: 13.42%-16.99%; medium: 17.00%-20.72%; high: 20.73%-30.10%; highest: more than 30.11%.

^5^ Percent of people born in a foreign country by county in 2000 from the Census 2000 ACS data. Lowest: less than 4.82%; low: 4.83%-9.98%; medium: 9.99%-18.64%; high: 18.65%-27.20%; highest: more than 27.21%.

NA- not reported due to small number of cases.

*Statistically significantly change in incidence from 2000 to 2016.

Supplementary table 2. Model with the interaction between ethnicity and year of diagnosis.

| **Variable** | **IRR (95%-CI)** |
| --- | --- |
| Ethnicity |  |
| *Hispanic all races* | Referent |
| *Non-Hispanic White* | 0.00 (0.00, 0.05) |
| *Non-Hispanic Black* | 0.00 (0.00, 0.00) |
| Year of diagnosis | 1.00 (0.99, 1.01) |
| Ethnicity × Year of diagnosis |  |
| *Hispanic all races × Year of diagnosis* | Referent |
| *Non-Hispanic White × Year of diagnosis* | 1.01 (1.00, 1.02)* |
| *Non-Hispanic Black × Year of diagnosis* | 1.02 (1.00, 1.03)* |

*Statistically significant IRR with p< 0.05.

Supplementary table 3: Age-Adjusted Incidence Rates (AAIR; SEER18, 2000-2016) for pilocytic astrocytomas by selected demographic characteristics

|  | **Overall** | | | **Non-Hispanic White** | | | **Hispanic all races** | | |
| --- | --- | --- | --- | --- | --- | --- | --- | --- | --- |
|  | **N** | **AAIR^1^**  **(95%-CI)** | **APC^2^**  **(95%-CI)** | **N** | **AAIR^1^**  **(95%-CI)** | **APC^2^**  **(95%-CI)** | **N** | **AAIR^1^**  **(95%-CI)** | **APC^2^**  **(95%-CI)** |
| **Overall** | 3,267 | 0.82  (0.79, 0.85) | 0.59  (-0.25, 1.43) | 1,993 | 1.06  (1.01, 1.11) | 0.91  (-0.21, 2.03) | 707 | 0.60  (0.56, 0.65) | -0.82  (-1.82, 0.19) |
| **Gender** |  |  |  |  |  |  |  |  |  |
| ***Male*** | 1,665 | 0.82  (0.78, 0.86) | 0.56  (-0.68, 1.81) | 1,028 | 1.06  (1.00, 1.13) | 0.92  (-0.74, 2.61) | 354 | 0.59  (0.53, 0.66) | -0.38  (-2, 1.28) |
| ***Female*** | 1,602 | 0.82  (0.78, 0.87) | 0.55  (-0.57, 1.69) | 965 | 1.05  (0.99, 1.12) | 1.01  (-0.68, 2.74) | 353 | 0.61  (0.55, 0.68) | -1.08  (-2.75, 0.63) |
| **Age (years)** |  |  |  |  |  |  |  |  |  |
| ***0-4*** | 969 | 1.00  (0.94, 1.06) | -0.19  (-1.60, 1.24) | 547 | 1.24  (1.13, 1.34) | 0.04  (-1.72, 1.84) | 244 | 0.79  (0.70, 0.90) | -1.73  (-3.98, 0.57) |
| ***5-9*** | 916 | 0.94  (0.88, 1.00) | 1.01  (-0.24, 2.29) | 553 | 1.20  (1.11, 1.31) | **1.97***  **(0.06, 3.91)** | 207 | 0.71  (0.61, 0.81) | -1.78  (-4.53, 1.06) |
| ***10-14*** | 816 | 0.80  (0.75, 0.86) | 0.72  (-0.75, 2.22) | 532 | 1.08  (0.99, 1.18) | 0.75  (-0.95, 2.49) | 147 | 0.51  (0.43, 0.6) | 1.78  (-1.18, 4.83) |
| ***15-19*** | 566 | 0.55  (0.51, 0.6) | 0.85  (-0.98, 2.71) | 361 | 0.72  (0.65, 0.80) | 0.97  (-1.87, 3.88) | 109 | 0.39  (0.32, 0.47) | -1.93  (-5.27, 1.53) |
| **Median household income in 2000^3^** |  |  |  |  |  |  |  |  |  |
| ***Lowest*** | 691 | 0.82  (0.76, 0.88) | 0.23  (-1.14, 1.61) | 468 | 1.04  (0.94, 1.13) | 0.74  (-1.36, 2.88) | 119 | 0.60  (0.50, 0.72) | -1.00  (-4.49, 2.62) |
| ***Low*** | 633 | 0.69  (0.64, 0.75) | 0.96  (-0.46, 2.41) | 291 | 0.98  (0.87, 1.10) | 1.75  (-0.71, 4.27) | 227 | 0.54  (0.47, 0.62) | -0.19  (-2.97, 2.66) |
| ***Medium*** | 632 | 0.87  (0.81, 0.94) | 0.70  (-0.55, 1.98) | 389 | 1.07  (0.97, 1.19) | 1.70  (-0.55, 4.00) | 150 | 0.71  (0.60, 0.83) | -2.08  (-5.27, 1.21) |
| ***High*** | 658 | 0.88  (0.81, 0.95) | 0.57  (-1.75, 2.94) | 425 | 1.09  (0.99, 1.20) | 0.40  (-1.49, 2.34) | 88 | 0.59  (0.47, 0.72) | -0.14  (-4.19, 4.07) |
| ***Highest*** | 653 | 0.87  (0.81, 0.94) | 0.09  (-1.56, 1.77) | 420 | 1.10  (0.99, 1.21) | 0.88  (-1.46, 3.27) | 123 | 0.62  (0.51, 0.73) | -0.87  (-3.86, 2.21) |
| **% < High school education in 2000^4^** |  |  |  |  |  |  |  |  |  |
| ***Lowest*** | 748 | 0.99  (0.92, 1.06) | 1.08  (-0.51, 2.69) | 556 | 1.09  (1.00, 1.19) | 1.14  (-0.62, 2.93) | 84 | 0.78  (0.62, 0.97) | 1.29  (-2.10, 4.79) |
| ***Low*** | 666 | 0.86  (0.80, 0.93) | -0.41  (-1.98, 1.19) | 438 | 1.13  (1.03, 1.24) | -0.12  (-2.05, 1.84) | 89 | 0.53  (0.43, 0.65) | -0.95  (-4.63, 2.87) |
| ***Medium*** | 752 | 0.81  (0.75, 0.87) | 1.12  (-0.92, 3.19) | 450 | 0.98  (0.89, 1.08) | 1.89  (-0.64, 4.47) | 161 | 0.62  (0.53, 0.72) | -1.35  (-4.70, 2.11) |
| ***High*** | 914 | 0.71  (0.66, 0.75) | 0.56  (-1.00, 2.14) | 445 | 1.02  (0.93, 1.12) | 0.88  (-1.39, 3.20) | 301 | 0.55  (0.49, 0.62) | -0.73  (-3.36, 1.98) |
| ***Highest*** | 311 | 0.81  (0.72, 0.91) | 0.74  (-0.92, 2.43) | 196 | 1.11  (0.96, 1.28) | 1.55  (-0.52, 3.66) | 86 | 0.62  (0.50, 0.77) | -0.86  (-5.03, 3.50) |
| **Percentage foreign-born in 2000^5^** |  |  |  |  |  |  |  |  |  |
| ***Lowest*** | 742 | 0.91  (0.84, 0.97) | 1.34  (-0.35, 3.06) | 588 | 1.05  (0.97, 1.14) | 1.43  (-0.43, 3.32) | 32 | 0.58  (0.40, 0.82) | NA |
| ***Low*** | 710 | 0.92  (0.85, 0.99) | 1.33  (-0.64, 3.34) | 506 | 1.04  (0.95, 1.13) | 1.16  (-0.84, 3.19) | 78 | 0.69  (0.54, 0.86) | NA |
| ***Medium*** | 647 | 0.80  (0.74, 0.86) | 0.94  (-1.38, 3.33) | 402 | 1.08  (0.98, 1.19) | 0.80  (-2.14, 3.83) | 129 | 0.52  (0.43, 0.61) | 1.11  (-2.22, 4.54) |
| ***High*** | 636 | 0.81  (0.75, 0.88) | -1.11  (-2.50, 0.30) | 282 | 1.07  (0.95, 1.20) | 1.21  (-1.34, 3.82) | 244 | 0.73  (0.64, 0.83) | **-3.59***  **(-5.67, -1.46)** |
| ***Highest*** | 532 | 0.67  (0.61, 0.73) | -0.62  (-2.44, 1.24) | 215 | 1.09  (0.95, 1.24) | -1.11  (-3.72, 1.58) | 224 | 0.53  (0.46, 0.60) | 0.23  (-2.37, 2.90) |
|  | **Non-Hispanic Black** | | | **Non-Hispanic API** | | | **Non-Hispanic AIAN** | | |
|  | **N** | **AAIR^1^**  **(95%-CI)** | **APC^2^**  **(95%-CI)** | **N** | **AAIR^1^**  **(95%-CI)** | **APC^2^**  **(95%-CI)** | **N** | **AAIR^1^**  **(95%-CI)** | **APC^2^**  **(95%-CI)** |
| **Overall** | 321 | 0.59  (0.53, 0.66) | 3.06  (-0.02, 6.24) | 170 | 0.50  (0.42, 0.58) | 0.28  (-2.56, 3.20) | 25 | 0.62  (0.40, 0.92) | NA |
| **Gender** |  |  |  |  |  |  |  |  |  |
| ***Male*** | 161 | 0.58  (0.50, 0.68) | 2.39  (-1.23, 6.13) | 86 | 0.49  (0.39, 0.61) | 1.32  (-3.11, 5.96) | 12 | 0.60  (0.31, 1.05) | NA |
| ***Female*** | 160 | 0.60  (0.51, 0.70) | **3.72***  **(0.34, 7.22)** | 84 | 0.50  (0.40, 0.62) | -0.71  (-5.04, 3.81) | 13 | 0.65  (0.35, 1.12) | NA |
| **Age (years)** |  |  |  |  |  |  |  |  |  |
| ***0-4*** | 96 | 0.75  (0.60, 0.91) | 2.31  (-2.22, 7.04) | 56 | 0.67  (0.50, 0.87) | -1.47  (-5.70, 2.95) | 8 | 0.86  (0.37, 1.70) | NA |
| ***5-9*** | 87 | 0.65  (0.52, 0.80) | 0.82  (-3.49, 5.33) | 52 | 0.62  (0.46, 0.81) | NA | 3 | 0.32  (0.07, 0.92) | NA |
| ***10-14*** | 85 | 0.60  (0.48, 0.74) | 2.03  (-2.75, 7.03) | 32 | 0.37  (0.25, 0.52) | NA | 10 | 0.96  (0.46, 1.76) | NA |
| ***15-19*** | 53 | 0.37  (0.28, 0.49) | NA | 30 | 0.34  (0.23, 0.48) | NA | 4 | 0.37  (0.10, 0.95) | NA |
| **Median household income in 2000^3^** |  |  |  |  |  |  |  |  |  |
| ***Lowest*** | 85 | 0.53  (0.43, 0.66) | 0.94  (-4.71, 6.92) | 8 | 0.40  (0.17, 0.79) | NA | 5 | 0.30  (0.10, 0.69) | NA |
| ***Low*** | 80 | 0.64  (0.51, 0.80) | 2.98  (-2.38, 8.62) | 27 | 0.35  (0.23, 0.51) | NA | 4 | 1.11  (0.30, 2.86) | NA |
| ***Medium*** | 62 | 0.64  (0.49, 0.82) | NA | 23 | 0.48  (0.30, 0.72) | NA | 3 | 0.54  (0.11, 1.57) | NA |
| ***High*** | 54 | 0.55  (0.41, 0.71) | NA | 57 | 0.58  (0.44, 0.75) | NA | 10 | 0.88  (0.42, 1.62) | NA |
| ***Highest*** | 40 | 0.64  (0.46, 0.88) | NA | 55 | 0.54  (0.41, 0.71) | NA | 3 | 1.33  (0.27, 3.98) | NA |
| **Percentage < high school education in 2000^4^** |  |  |  |  |  |  |  |  |  |
| ***Lowest*** | 42 | 0.61  (0.44, 0.82) | NA | 37 | 0.62  (0.43, 0.85) | NA | 17 | 1.33  (0.78, 2.13) | NA |
| ***Low*** | 67 | 0.64  (0.50, 0.81) | 3.68  (-1.82, 9.48) | 50 | 0.45  (0.34, 0.60) | -1.47  (-6.19, 3.49) | 4 | 0.59  (0.16, 1.50) | NA |
| ***Medium*** | 73 | 0.64  (0.50, 0.81) | NA | 52 | 0.56  (0.41, 0.73) | 1.20  (-3.71, 6.35) | 0 | 0.00  (0.00, 0.70) | NA |
| ***High*** | 124 | 0.56  (0.46, 0.66) | 3.60  (-0.41, 7.76) | 34 | 0.41  (0.28, 0.57) | NA | 4 | 0.42  (0.11, 1.08) | NA |
| ***Highest*** | 25 | 0.50  (0.32, 0.74) | NA | 2 | 0.17  (0.02, 0.61) | NA | 0 | 0.00  (0.00, 0.58) | NA |
| **Percentage foreign-born in 2000^5^** |  |  |  |  |  |  |  |  |  |
| ***Lowest*** | 97 | 0.55  (0.44, 0.67) | 2.56  (-3.47, 8.97) | 10 | 0.86  (0.41, 1.59) | NA | 2 | 0.15  (0.02, 0.53) | NA |
| ***Low*** | 86 | 0.67  (0.54, 0.83) | 3.14  (-1.38, 7.87) | 10 | 0.30  (0.15, 0.56) | NA | 21 | 1.43  (0.88, 2.19) | NA |
| ***Medium*** | 68 | 0.62  (0.48, 0.78) | NA | 34 | 0.46  (0.32, 0.65) | NA | 1 | 0.22  (0.01, 1.19) | NA |
| ***High*** | 32 | 0.44  (0.30, 0.62) | NA | 65 | 0.60  (0.46, 0.77) | NA | 1 | 0.23  (0.01, 1.38) | NA |
| ***Highest*** | 38 | 0.70  (0.49, 0.96) | NA | 51 | 0.44  (0.33, 0.58) | -2.46  (-7.41, 2.77) | 0 | 0.00  (0.00, 1.69) | NA |
|  |  |  |  |  |  |  |  |  |  |

Supplementary table 3 legend:

^1^ AAIR per 100,000 persons, diagnosed from 2000-2016, SEER18 data.

^2^ Annual percent change (APC) for 2000-2016 using SEER18 data.

^3^ Median household income by county in 2000 from the Census 2000 ACS data. Lowest: less than $39,010; low: $39,011-$42,190; medium: $42,191-$47,320; high: $47,321-$57,310; highest: more than $57,311.

^4^ Percent of people less than high school education by county in 2000 from the Census 2000 ACS data. Lowest: less than 13.41%; low: 13.42%-16.99%; medium: 17.00%-20.72%; high: 20.73%-30.10%; highest: more than 30.11%.

^5^ Percent of people born in a foreign country by county in 2000 from the Census 2000 ACS data. Lowest: less than 4.82%; low: 4.83%-9.98%; medium: 9.99%-18.64%; high: 18.65%-27.20%; highest: more than 27.21%.

NA- not reported due to small number of cases.

*Statistically significantly change in incidence from 2000 to 2016.

Supplementary table 4: Age-Adjusted Incidence Rates (AAIR; SEER18, 2000-2016) for gliomas by selected demographic characteristics in California.

|  | **Overall** | | | **Non-Hispanic White** | | | **Hispanic all races** | | |
| --- | --- | --- | --- | --- | --- | --- | --- | --- | --- |
|  | **N** | **AAIR^1^**  **(95%-CI)** | **APC^2^**  **(95%-CI)** | **N** | **AAIR^1^**  **(95%-CI)** | **APC^2^**  **(95%-CI)** | **N** | **AAIR^1^**  **(95%-CI)** | **APC^2^**  **(95%-CI)** |
| **Overall** | 3,644 | 2.08  (2.01, 2.15) | 0.46  (-0.36, 1.27) | 1,532 | 2.75  (2.61, 2.89) | **1.29***  **(0.36, 2.23)** | 1496 | 1.73  (1.65, 1.82) | -0.15  (-1.66, 1.37) |
| **Gender** |  |  |  |  |  |  |  |  |  |
| ***Male*** | 1,902 | 2.12  (2.02, 2.21) | 0.40  (-0.64, 1.45) | 818 | 2.85  (2.65, 3.05) | 1.22  (-0.27, 2.74) | 748 | 1.70  (1.58, 1.83) | -0.74  (-2.75, 1.31) |
| ***Female*** | 1,742 | 2.04  (1.94, 2.14) | 0.44  (-0.55, 1.44) | 714 | 2.64  (2.45, 2.84) | **1.33***  **(0.07, 2.61)** | 748 | 1.77  (1.64, 1.90) | 0.33  (-1.16, 1.84) |
| **Age (years)** |  |  |  |  |  |  |  |  |  |
| ***0-4*** | 1,059 | 2.47  (2.33, 2.63) | 0.33  (-0.83, 1.50) | 403 | 3.12  (2.82, 3.44) | 1.93  (-0.17, 4.09) | 473 | 2.15  (1.96, 2.35) | -0.78  (-2.64, 1.12) |
| ***5-9*** | 1,026 | 2.39  (2.25, 2.54) | 0.14  (-1.26, 1.55) | 409 | 3.08  (2.79, 3.39) | 0.95  (-1.05, 3.00) | 453 | 2.11  (1.92, 2.31) | -1.06  (-3.74, 1.70) |
| ***10-14*** | 831 | 1.86  (1.73, 1.99) | 1.01  (-0.50, 2.55) | 386 | 2.64  (2.38, 2.91) | 1.39  (-0.37, 3.19) | 313 | 1.46  (1.30, 1.63) | 0.68  (-2.09, 3.53) |
| ***15-19*** | 728 | 1.61  (1.49, 1.73) | 0.45  (-1.69, 2.62) | 334 | 2.17  (1.94, 2.41) | 0.80  (-1.90, 3.57) | 257 | 1.24  (1.09, 1.40) | 0.84  (-2.16, 3.94) |
| **Median household income in 2000^3^** |  |  |  |  |  |  |  |  |  |
| ***Lowest*** | 432 | 2.02  (1.83, 2.22) | -0.21  (2.10, 1.72) | 182 | 2.51  (2.16, 2.90) | 0.53  (-1.65, 2.77) | 211 | 1.81  (1.57, 2.07) | -1.01  (-3.78, 1.83) |
| ***Low*** | 1,242 | 1.90  (1.80, 2.01) | -0.30  (-1.63, 1.04) | 410 | 2.77  (2.51, 3.05) | -0.71  (-2.13, 0.74) | 618 | 1.61  (1.49, 1.75) | -0.07  (-2.03, 1.93) |
| ***Medium*** | 779 | 2.23  (2.07, 2.39) | 1.07  (-0.66, 2.83) | 363 | 2.71  (2.44, 3.01) | **3.39***  **(0.91, 5.93)** | 310 | 1.98  (1.77, 2.21) | -1.47  (-4.26, 1.41) |
| ***High*** | 370 | 2.15  (1.94, 2.38) | -0.23  (-2.35, 1.93) | 172 | 2.82  (2.41, 3.27) | 0.73  (-2.07, 3.61) | 105 | 1.68  (1.37, 2.04) | -1.33  (-6.37, 3.98) |
| ***Highest*** | 821 | 2.25  (2.10, 2.41) | 1.57  (-0.05, 3.23) | 405 | 2.87  (2.59, 3.16) | 2.31  (-0.38, 5.08) | 252 | 1.75  (1.54, 1.98) | 1.58  (-1.19, 4.42) |
| **Percentage < high school education in 2000^4^** |  |  |  |  |  |  |  |  |  |
| ***Lowest*** | 228 | 2.68  (2.34, 3.05) | 0.34  (-2.30, 3.05) | 142 | 3.00  (2.53, 3.54) | 0.63  (-3.18, 4.60) | 37 | 1.69  (1.19, 2.32) | NA |
| ***Low*** | 582 | 2.25  (2.07, 2.44) | 0.99  (-0.90, 2.92) | 332 | 3.04  (2.72, 3.39) | 1.18  (-0.65, 3.05) | 136 | 1.65  (1.38, 1.95) | 0.87  (-3.48, 5.43) |
| ***Medium*** | 1,133 | 2.17  (2.05, 2.30) | **1.71***  **(0.34, 3.10)** | 532 | 2.62  (2.40, 2.85) | **3.46***  **(1.54, 5.42)** | 390 | 1.91  (1.73, 2.11) | 0.49  (-2.11, 3.15) |
| ***High*** | 1,519 | 1.93  (1.83, 2.03) | -0.35  (-1.63, 0.94) | 526 | 2.74  (2.51, 2.99) | -0.22  (-1.64, 1.23) | 751 | 1.63  (1.52, 1.76) | -0.20  (-2.16, 1.80) |
| ***Highest*** | 366 | 1.96  (1.77, 2.18) | -0.18  (-2.22, 1.91) | 114 | 2.50  (2.06, 3.00) | 0.37  (-2.69, 3.54) | 221 | 1.83  (1.60, 2.09) | -0.90  (-4.02, 2.32) |
| **Percentage foreign-born in 2000^5^** |  |  |  |  |  |  |  |  |  |
| ***Lowest*** | 57 | 2.31  (1.75, 3.00) | -2.89  (-8.29, 2.82) | 44 | 2.34  (1.70, 3.15) | NA | 7 | 1.95  (0.78, 4.04) | NA |
| ***Low*** | 140 | 2.71  (2.27, 3.19) | -1.26  (-3.74, 1.29) | 113 | 3.19  (2.62, 3.84) | 0.01  (-3.54, 3.69) | 17 | 1.45  (0.84, 2.32) | NA |
| ***Medium*** | 702 | 2.10  (1.95, 2.27) | 0.83  (-0.99, 2.68) | 356 | 2.95  (2.65, 3.27) | 1.44  (-0.77, 3.69) | 250 | 1.59  (1.40, 1.80) | -0.29  (-3.33, 2.84) |
| ***High*** | 1,235 | 2.15  (2.03, 2.28) | 0.73  (-0.37, 1.84) | 502 | 2.63  (2.41, 2.88) | **3.11***  **(1.06, 5.20)** | 530 | 1.91  (1.75, 2.08) | -0.97  (-2.76, 0.85) |
| ***Highest*** | 1,510 | 1.96  (1.86, 2.06) | 0.11  (-1.40, 1.65) | 517 | 2.69  (2.47, 2.94) | -0.10  (-1.77, 1.60) | 692 | 1.67  (1.55, 1.80) | 0.45  (-1.63, 2.57) |
|  | **Non-Hispanic Black** | | | **Non-Hispanic API** | | | **Non-Hispanic AIAN** | | |
|  | **N** | **AAIR^1^**  **(95%-CI)** | **APC^2^**  **(95%-CI)** | **N** | **AAIR^1^**  **(95%-CI)** | **APC^2^**  **(95%-CI)** | **N** | **AAIR^1^**  **(95%-CI)** | **APC^2^**  **(95%-CI)** |
| **Overall** | 221 | 1.83  (1.60, 2.09) | 2.18  (-1.16, 5.62) | 339 | 1.66  (1.48, 1.84) | 0.79  (-1.54, 3.18) | 13 | 1.19  (0.63, 2.06) | NA |
| **Gender** |  |  |  |  |  |  |  |  |  |
| ***Male*** | 120 | 1.95  (1.61, 2.33) | **5.60***  **(1.04, 10.37)** | 192 | 1.82  (1.57, 2.10) | 1.39  (-1.43, 4.30) | 6 | 1.11  (0.40, 2.44) | NA |
| ***Female*** | 101 | 1.71  (1.40, 2.08) | -1.52  (-5.70, 2.85) | 147 | 1.48  (1.25, 1.74) | 0.00  (-3.00, 3.11) | 7 | 1.28  (0.51, 2.69) | NA |
| **Age (years)** |  |  |  |  |  |  |  |  |  |
| ***0-4*** | 68 | 2.47  (1.91, 3.13) | 0.64  (-4.14, 5.65) | 97 | 1.98  (1.61, 2.42) | 0.22  (-3.64, 4.24) | 2 | 0.90  (0.11, 3.26) | NA |
| ***5-9*** | 58 | 1.99  (1.51, 2.57) | 0.92  (-5.01, 7.23) | 95 | 1.92  (1.56, 2.35) | NA | 1 | 0.41  (0.01, 2.31) | NA |
| ***10-14*** | 51 | 1.58  (1.17, 2.07) | NA | 70 | 1.34  (1.05, 1.69) | 2.92  (-1.04, 7.03) | 1 | 0.37  (0.01, 2.04) | NA |
| ***15-19*** | 44 | 1.33  (0.97, 1.78) | NA | 77 | 1.39  (1.10, 1.74) | 0.14  (-4.40, 4.89) | 9 | 3.09  (1.41, 5.87) | NA |
| **Median household income in 2000^3^** |  |  |  |  |  |  |  |  |  |
| ***Lowest*** | 13 | 1.50  (0.80, 2.57) | NA | 18 | 1.50  (0.89, 2.39) |  | 5 | 1.41  (0.45, 3.30) | NA |
| ***Low*** | 108 | 1.89  (1.55, 2.28) | 1.49  (-2.92, 6.10) | 99 | 1.55  (1.26, 1.89) | -0.25  (-5.20, 4.96) | 1 | 0.38  (0.01, 2.38) | NA |
| ***Medium*** | 45 | 1.72  (1.25, 2.30) | NA | 53 | 1.67  (1.25, 2.19) | NA | 2 | 0.76  (0.09, 2.90) | NA |
| ***High*** | 33 | 2.08  (1.43, 2.92) | NA | 50 | 1.59  (1.18, 2.10) | -0.26  (-4.28, 3.92) | 2 | 1.90  (0.23, 7.39) | NA |
| ***Highest*** | 22 | 1.73  (1.08, 2.62) | NA | 119 | 1.80  (1.49, 2.15) | 2.11  (-1.93, 6.33) | 3 | 2.31  (0.47, 6.93) | NA |
| **% < High school education in 2000^4^** |  |  |  |  |  |  |  |  |  |
| ***Lowest*** | 10 | 1.62  (0.78, 2.99) | NA | 25 | 2.77  (1.79, 4.10) | NA | 1 | 1.72  (0.04, 10.4) | NA |
| ***Low*** | 25 | 1.48  (0.95, 2.18) | NA | 77 | 1.59  (1.26, 1.99) | NA | 6 | 2.36  (0.86, 5.22) | NA |
| ***Medium*** | 67 | 1.99  (1.54, 2.53) | NA | 124 | 1.58  (1.32, 1.89) | -0.38  (-3.26, 2.58) | 5 | 1.38  (0.45, 3.29) | NA |
| ***High*** | 120 | 1.84  (1.52, 2.20) | 0.90  (-3.50, 5.51) | 110 | 1.57  (1.29, 1.89) | -1.05  (-5.59, 3.72) | 3 | 0.83  (0.17, 2.48) | NA |
| ***Highest*** | 14 | 1.77  (0.97, 2.96) | NA | 17 | 1.62  (0.94, 2.60) | NA | 0 | 0.00  (0.00, 2.7) | NA |
| **Percentage oreign-born in 2000^5^** |  |  |  |  |  |  |  |  |  |
| ***Lowest*** | 1 | 2.01  (0.05, 11.44) | NA | 0 | 0.00  (0.00, 6.34) | NA | 2 | 1.82  (0.22, 6.59) | NA |
| ***Low*** | 2 | 1.95  (0.24, 7.06) | NA | 5 | 1.95  (0.62, 4.62) | NA | 2 | 1.75  (0.21, 6.54) | NA |
| ***Medium*** | 47 | 1.63  (1.20, 2.17) | NA | 34 | 1.32  (0.91, 1.84) | NA | 5 | 1.84  (0.59, 4.40) | NA |
| ***High*** | 68 | 1.71  (1.32, 2.16) | 0.09  (-3.47, 3.79) | 115 | 1.84  (1.52, 2.21) | 2.09  (-2.57, 6.98) | 2 | 0.52  (0.06, 2.00) | NA |
| ***Highest*** | 103 | 2.04  (1.66, 2.47) | 0.92  (-4.14, 6.25) | 185 | 1.63  (1.40, 1.88) | 0.42  (-3.03, 4.00) | 2 | 0.89  (0.11, 3.29) | NA |
|  |  |  |  |  |  |  |  |  |  |

Supplementary table 4 legend:

^1^ AAIR per 100,000 persons, diagnosed from 2000-2016, SEER18 data.

^2^ Annual percent change (APC) for 2000-2016 using SEER18 data.

^3^ Median household income by county in 2000 from the Census 2000 ACS data. Lowest: less than $39,010; low: $39,011-$42,190; medium: $42,191-$47,320; high: $47,321-$57,310; highest: more than $57,311.

^4^ Percent of people less than high school education by county in 2000 from the Census 2000 ACS data. Lowest: less than 13.41%; low: 13.42%-16.99%; medium: 17.00%-20.72%; high: 20.73%-30.10%; highest: more than 30.11%.

^5^ Percent of people born in a foreign country by county in 2000 from the Census 2000 ACS data. Lowest: less than 4.82%; low: 4.83%-9.98%; medium: 9.99%-18.64%; high: 18.65%-27.20%; highest: more than 27.21%.

NA- not reported due to small number of cases.

*Statistically significantly change in incidence from 2000 to 2016.

Supplementary table 5: Age-Adjusted Incidence Rates (AAIR; SEER18, 2000-2016) for medulloblastomas by selected demographic characteristics

|  | **Overall** | | | **Non-Hispanic White** | | | **Hispanic all races** | | |
| --- | --- | --- | --- | --- | --- | --- | --- | --- | --- |
|  | **N** | **AAIR^1^**  **(95%-CI)** | **APC^2^**  **(95%-CI)** | **N** | **AAIR^1^**  **(95%-CI)** | **APC^2^**  **(95%-CI)** | **N** | **AAIR^1^**  **(95%-CI)** | **APC^2^**  **(95%-CI)** |
| **Overall** | 1,552 | 0.39  (0.37, 0.41) | 0.86  (-0.10, 1.84) | 887 | 0.48  (0.44, 0.51) | **1.52***  **(0.15, 2.91)** | 411 | 0.34  (0.31, 0.38) | -1.46  (-3.08, 0.19) |
| **Gender** |  |  |  |  |  |  |  |  |  |
| ***Male*** | 981 | 0.48  (0.45, 0.51) | 1.07  (-0.07, 2.22) | 579 | 0.61  (0.56, 0.66) | **1.90***  **(0.08, 3.76)** | 257 | 0.42  (0.37, 0.48) | -1.72  (-3.63, 0.23) |
| ***Female*** | 571 | 0.29  (0.27, 0.32) | 0.47  (-1.58, 2.55) | 308 | 0.34  (0.30, 0.38) | 0.95  (-1.52, 3.48) | 154 | 0.26  (0.22, 0.31) | -1.40  (-4.95, 2.28) |
| **Age (years)** |  |  |  |  |  |  |  |  |  |
| ***0-4*** | 529 | 0.54  (0.50, 0.59) | -0.38  (-1.43, 0.67) | 283 | 0.64  (0.57, 0.72) | 0.61  (-1.44, 2.70) | 167 | 0.54  (0.46, 0.63) | -2.20  (-4.71, 0.37) |
| ***5-9*** | 569 | 0.58  (0.53, 0.63) | 1.76  (-0.02, 3.57) | 317 | 0.69  (0.62, 0.77) | **2.44***  **(0.37, 4.55)** | 148 | 0.50  (0.43, 0.59) | -1.42  (-4.42, 1.67) |
| ***10-14*** | 279 | 0.27  (0.24, 0.31) | 2.10  (-0.36, 4.61) | 178 | 0.36  (0.31, 0.42) | 2.17  (-0.97, 5.40) | 56 | 0.20  (0.15, 0.25) | 1.06  (-3.67, 6.02) |
| ***15-19*** | 175 | 0.17  (0.15, 0.20) | 0.22  (-2.67, 3.19) | 109 | 0.22  (0.18, 0.26) | 1.06  (-2.95, 5.23) | 40 | 0.14  (0.10, 0.20) | NA |
| **Median household income in 2000^3^** |  |  |  |  |  |  |  |  |  |
| ***Lowest*** | 312 | 0.37  (0.33, 0.41) | -0.25  (-3.01, 2.59) | 201 | 0.45  (0.39, 0.52) | -0.73  (-3.51, 2.12) | 58 | 0.29  (0.22, 0.38) | NA |
| ***Low*** | 311 | 0.34  (0.30, 0.38) | 1.98  (-0.68, 4.72) | 131 | 0.45  (0.37, 0.53) | **5.65***  **(1.57, 9.90)** | 140 | 0.33  (0.28, 0.39) | -0.85  (-4.83, 3.30) |
| ***Medium*** | 309 | 0.43  (0.38, 0.48) | 0.99  (-0.75, 2.76) | 183 | 0.51  (0.44, 0.59) | 1.48  (-1.22, 4.25) | 80 | 0.38  (0.30, 0.47) | -4.48  (-6.41, -2.51) |
| ***High*** | 295 | 0.39  (0.35, 0.44) | 0.61  (-1.5,0 2.77) | 181 | 0.47  (0.40, 0.54) | 0.60  (-1.67, 2.93) | 46 | 0.30  (0.22, 0.40) | -0.40  (-6.23, 5.79) |
| ***Highest*** | 325 | 0.44  (0.39, 0.49) | 0.97  (-1.49, 3.49) | 191 | 0.50  (0.44, 0.58) | 1.88  (-1.31, 5.18) | 87 | 0.43  (0.34, 0.53) | -1.85  (-6.83, 3.41) |
| **Percentage < high school education in 2000^4^** |  |  |  |  |  |  |  |  |  |
| ***Lowest*** | 335 | 0.44  (0.40, 0.49) | 0.67  (-1.41, 2.80) | 254 | 0.50  (0.44, 0.57) | 0.66  (-2.04, 3.43) | 42 | 0.38  (0.27, 0.51) | NA |
| ***Low*** | 326 | 0.42  (0.38, 0.47) | 0.10  (-1.98, 2.23) | 173 | 0.45  (0.39, 0.53) | 0.97  (-1.87, 3.89) | 73 | 0.43  (0.34, 0.54) | -4.48  (-9.59, 0.91) |
| ***Medium*** | 360 | 0.39  (0.35, 0.43) | 1.73  (-0.40, 3.90) | 207 | 0.46  (0.40, 0.52) | 2.67  (0.05, 5.35) | 95 | 0.36  (0.29, 0.44) | -1.13  (-5.22, 3.13) |
| ***High*** | 446 | 0.35  (0.32, 0.38) | 0.88  (-1.66, 3.50) | 202 | 0.47  (0.41, 0.54) | 2.33  (-1.65, 6.47) | 168 | 0.31  (0.26, 0.36) | -2.09  (-5.00, 0.91) |
| ***Highest*** | 148 | 0.39  (0.33, 0.45) | -1.41  (-5.16, 2.49) | 85 | 0.48  (0.39, 0.60) | -1.69  (-5.81, 2.61) | 50 | 0.36  (0.26, 0.47) | 1.85  (-4.35, 8.45) |
| **Percentage foreign-born in 2000^5^** |  |  |  |  |  |  |  |  |  |
| ***Lowest*** | 337 | 0.41  (0.37, 0.46) | 0.63  (-1.92, 3.25) | 267 | 0.48  (0.42, 0.54) | 1.01  (-1.97, 4.08) | 17 | 0.29  (0.17, 0.47) | NA |
| ***Low*** | 308 | 0.40  (0.36, 0.45) | 1.66  (-1.34, 4.76) | 220 | 0.46  (0.40, 0.52) | 2.19  (-2.12, 6.70) | 36 | 0.31  (0.22, 0.43) | NA |
| ***Medium*** | 332 | 0.41  (0.37, 0.46) | 0.41  (-1.87, 2.73) | 173 | 0.47  (0.40, 0.54) | -0.28  (-3.33, 2.87) | 112 | 0.44  (0.37, 0.53) | -0.15  (-4.55, 4.45) |
| ***High*** | 290 | 0.37  (0.33, 0.42) | -0.04  (-2.86, 2.86) | 125 | 0.48  (0.40, 0.57) | 1.28  (-2.37, 5.07) | 106 | 0.31  (0.26, 0.38) | -2.28  (-6.13, 1.72) |
| ***Highest*** | 285 | 0.36  (0.32, 0.40) | **1.99***  **(0.93, 3.06)** | 102 | 0.52  (0.43, 0.64) | **4.51***  **(1.09, 8.06)** | 140 | 0.33  (0.27, 0.38) | -1.92  (-4.50, 0.73) |
|  | **Non-Hispanic Black** | | | **Non-Hispanic API** | | | **Non-Hispanic AIAN** | | |
|  | **N** | **AAIR^1^**  **(95%-CI)** | **APC^2^**  **(95%-CI)** | **N** | **AAIR^1^**  **(95%-CI)** | **APC^2^**  **(95%-CI)** | **N** | **AAIR^1^**  **(95%-CI)** | **APC^2^**  **(95%-CI)** |
| **Overall** | 120 | 0.22  (0.18, 0.27) | 2.01  (-1.50, 5.63) | 115 | 0.34  (0.28, 0.41) | 3.66  (-0.47, 7.97) | 13 | 0.33  (0.18, 0.57) | NA |
| **Gender** |  |  |  |  |  |  |  |  |  |
| ***Male*** | 60 | 0.22  (0.17, 0.28) | NA | 73 | 0.42  (0.33, 0.53) | **4.76***  **(1.27, 8.38)** | 8 | 0.41  (0.17, 0.80) | NA |
| ***Female*** | 60 | 0.23  (0.17, 0.29) | NA | 42 | 0.25  (0.18, 0.34) | NA | 5 | 0.26  (0.08, 0.61) | NA |
| **Age (years)** |  |  |  |  |  |  |  |  |  |
| ***0-4*** | 32 | 0.25  (0.17, 0.35) | NA | 41 | 0.49  (0.35, 0.66) | NA | 3 | 0.32  (0.07, 0.94) | NA |
| ***5-9*** | 49 | 0.37  (0.27, 0.49) | NA | 47 | 0.56  (0.41, 0.74) | NA | 7 | 0.74  (0.30, 1.52) | NA |
| ***10-14*** | 24 | 0.17  (0.11, 0.25) | NA | 20 | 0.23  (0.14, 0.36) | NA | 0 | 0.00  (0.00, 0.35) | NA |
| ***15-19*** | 15 | 0.11  (0.06, 0.17) | NA | 7 | 0.08  (0.03, 0.16) | NA | 3 | 0.28  (0.06, 0.81) | NA |
| **Median household income in 2000^3^** |  |  |  |  |  |  |  |  |  |
| ***Lowest*** | 40 | 0.25  (0.18, 0.34) | 0.60  (-3.41, 4.77) | 5 | 0.27  (0.09, 0.62) | NA | 6 | 0.36  (0.13, 0.77) | NA |
| ***Low*** | 22 | 0.18  (0.11, 0.27) | NA | 17 | 0.23  (0.13, 0.36) | NA | 1 | 0.30  (0.01, 1.64) | NA |
| ***Medium*** | 18 | 0.18  (0.11, 0.29) | NA | 25 | 0.53  (0.34, 0.78) | NA | 2 | 0.35  (0.04, 1.26) | NA |
| ***High*** | 28 | 0.28  (0.19, 0.41) | NA | 35 | 0.36  (0.25, 0.50) | NA | 3 | 0.28  (0.06, 0.80) | NA |
| ***Highest*** | 12 | 0.20  (0.10, 0.34) | NA | 33 | 0.32  (0.22, 0.45) | NA | 1 | 0.50  (0.01, 2.76) | NA |
| **% < high school education in 2000^4^** |  |  |  |  |  |  |  |  |  |
| ***Lowest*** | 13 | 0.19  (0.10, 0.32) | NA | 21 | 0.35  (0.22, 0.53) | NA | 3 | 0.25  (0.05, 0.72) | NA |
| ***Low*** | 28 | 0.27  (0.18, 0.39) | NA | 47 | 0.42  (0.31, 0.56) | -0.27  (-5.89, 5.69) | 5 | 0.74  (0.24, 1.73) | NA |
| ***Medium*** | 26 | 0.23  (0.15, 0.33) | NA | 27 | 0.29  (0.19, 0.42) | NA | 3 | 0.58  (0.12, 1.68) | NA |
| ***High*** | 51 | 0.23  (0.17, 0.31) | NA | 24 | 0.30  (0.19, 0.44) | NA | 1 | 0.10  (0.00, 0.56) | NA |
| ***Highest*** | 7 | 0.14  (0.06, 0.29) | NA | 2 | 0.18  (0.02, 0.65) | NA | 2 | 0.30  (0.04, 1.11) | NA |
| **Percentage foreign-born in 2000^5^** |  |  |  |  |  |  |  |  |  |
| ***Lowest*** | 43 | 0.24  (0.18, 0.33) | 0.79  (-4.27, 6.11) | 5 | 0.44  (0.14, 1.02) | NA | 4 | 0.29  (0.08, 0.73) | NA |
| ***Low*** | 31 | 0.24  (0.17, 0.35) | NA | 14 | 0.42  (0.23, 0.71) | NA | 5 | 0.36  (0.12, 0.84) | NA |
| ***Medium*** | 23 | 0.21  (0.13, 0.31) | NA | 20 | 0.27  (0.17, 0.42) | NA | 3 | 0.58  (0.12, 1.72) | NA |
| ***High*** | 14 | 0.19  (0.11, 0.33) | NA | 43 | 0.40  (0.29, 0.54) | NA | 1 | 0.28  (0.01, 1.51) | NA |
| ***Highest*** | 9 | 0.17  (0.08, 0.32) | NA | 33 | 0.29  (0.20, 0.40) | NA | 0 | 0.00  (0.00, 1.69) | NA |

Supplementary table 5 legend:

^1^ AAIR per 100,000 persons, diagnosed from 2000-2016, SEER18 data.

^2^ Annual percent change (APC) for 2000-2016 using SEER18 data.

^3^ Median household income by county in 2000 from the Census 2000 ACS data. Lowest: less than $39,010; low: $39,011-$42,190; medium: $42,191-$47,320; high: $47,321-$57,310; highest: more than $57,311.

^4^ Percent of people less than high school education by county in 2000 from the Census 2000 ACS data. Lowest: less than 13.41%; low: 13.42%-16.99%; medium: 17.00%-20.72%; high: 20.73%-30.10%; highest: more than 30.11%.

^5^ Percent of people born in a foreign country by county in 2000 from the Census 2000 ACS data. Lowest: less than 4.82%; low: 4.83%-9.98%; medium: 9.99%-18.64%; high: 18.65%-27.20%; highest: more than 27.21%.

NA- not reported due to small number of cases.

*Statistically significantly change in incidence from 2000 to 2016.

Supplementary figure 1a: Multivariate Poisson regression model of age-adjusted incidence rates (AAIR) among non-Hispanic White glioma cases.


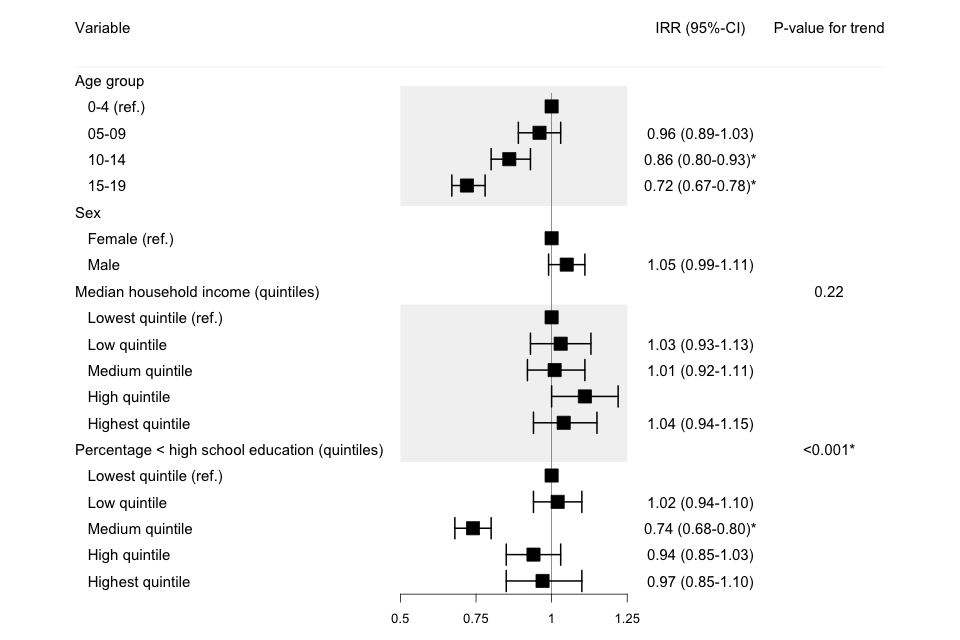


Supplementary figure 1b: Multivariate Poisson regression model of age-adjusted incidence rates (AAIR) among non-Hispanic Black glioma cases.


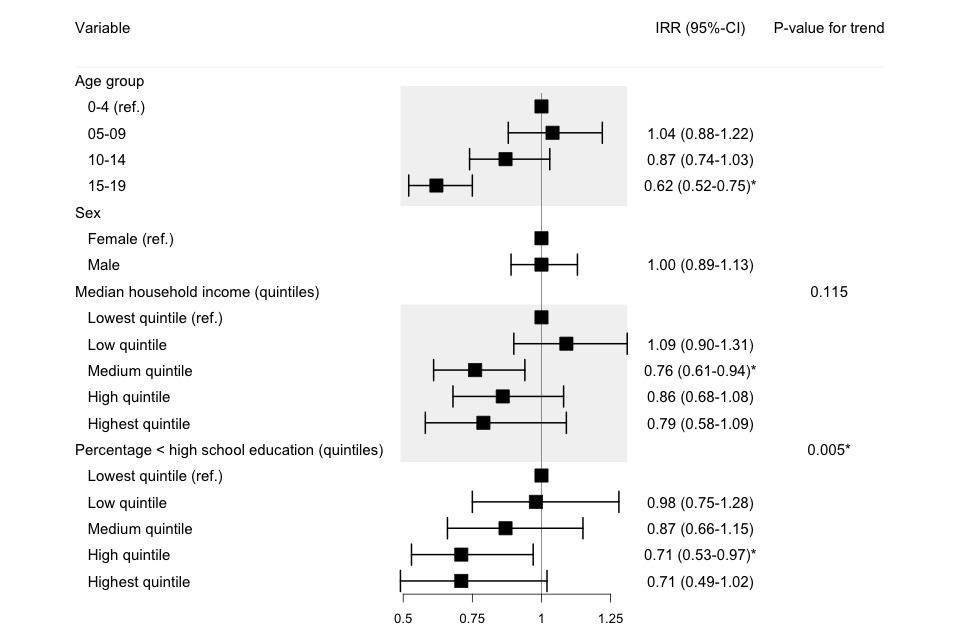


Supplementary figure 1c: Multivariate Poisson regression model of age-adjusted incidence rates (AAIR) among Hispanic glioma cases.


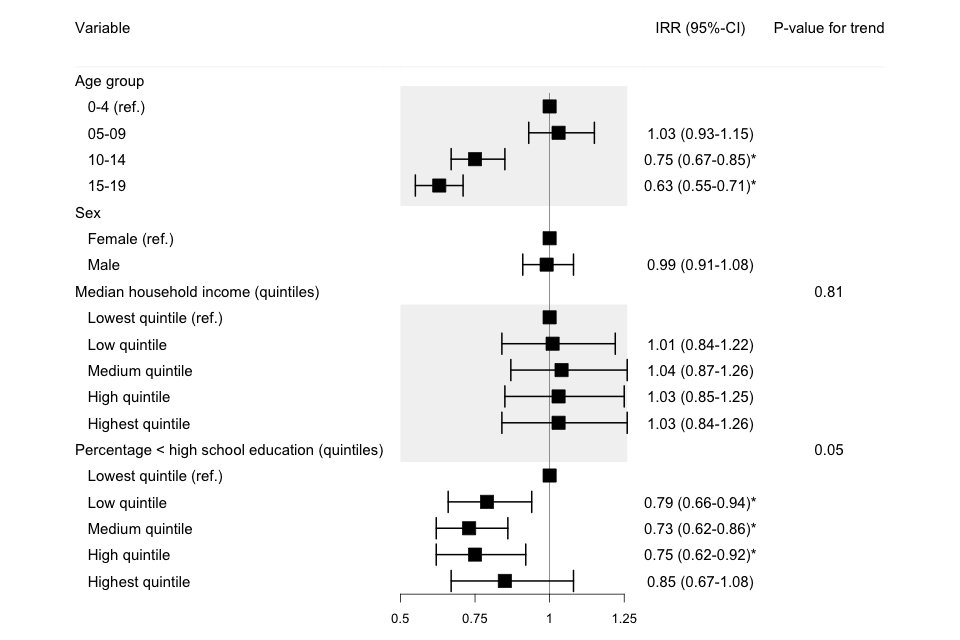


Supplementary figure 1d: Multivariate Poisson regression model of age-adjusted incidence rates (AAIR) among Hispanic glioma cases with percentage foreign-born.


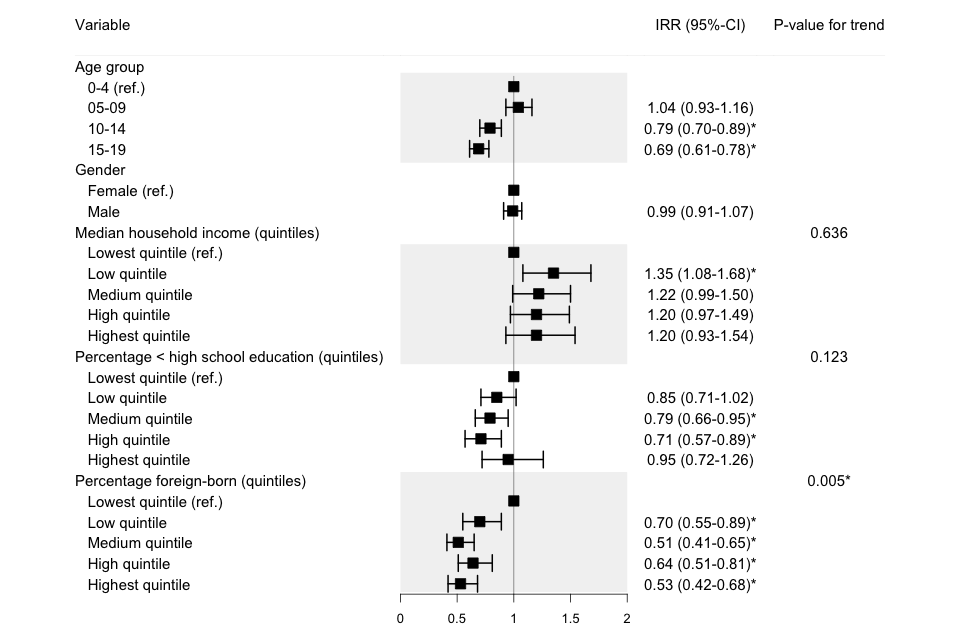


Supplementary figures 1a, 1b, 1c, and 1d legends: Figure 1a, 1b, and 1c are forest plots that depict incidence rate ratios (IRRs) for various risk factors and association with glioma for non-Hispanic Whites, non-Hispanic Blacks, and Hispanics, respectively. Figure 1d also depicts the IIRs for Hispanics but with percentage foreign-born as an additional variable in the model. IRRs were derived from a Poisson regression model using age-adjusted incidence rates (AAIR) as the dependent variable. Percentage foreign-born was included in the model for Hispanics. Standard population in each category and total standard population were derived from the Census 2000 ACS data.

*Statistically significant incidence rate ratio (IRR).

Supplementary figure 2a: Multivariate Poisson regression model of age-adjusted incidence rates (AAIR) among non-Hispanic White medulloblastoma cases.


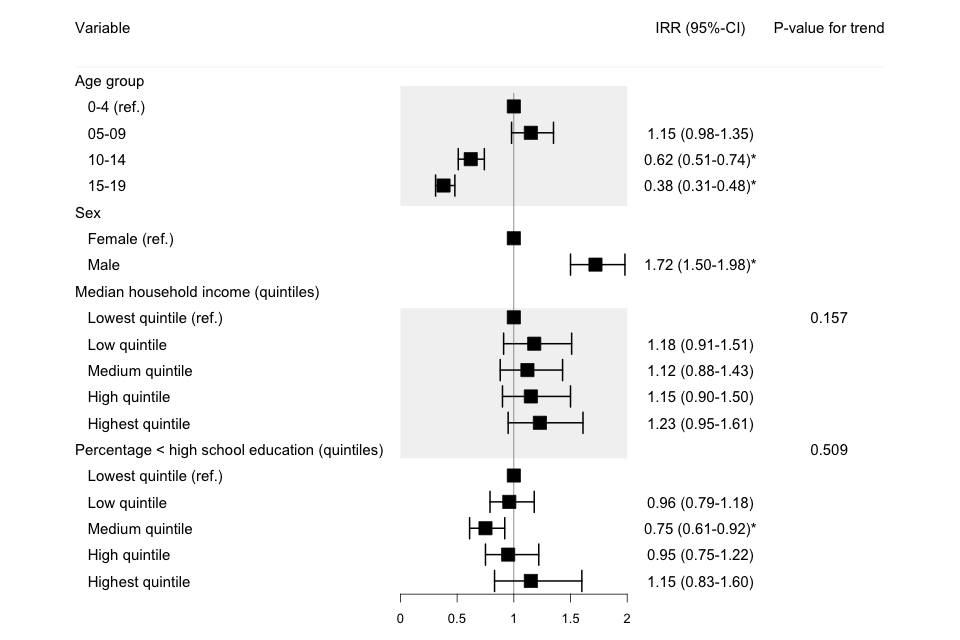


Supplementary figure 2b: Multivariate Poisson regression model of age-adjusted incidence rates (AAIR) among non-Hispanic Black medulloblastoma cases.


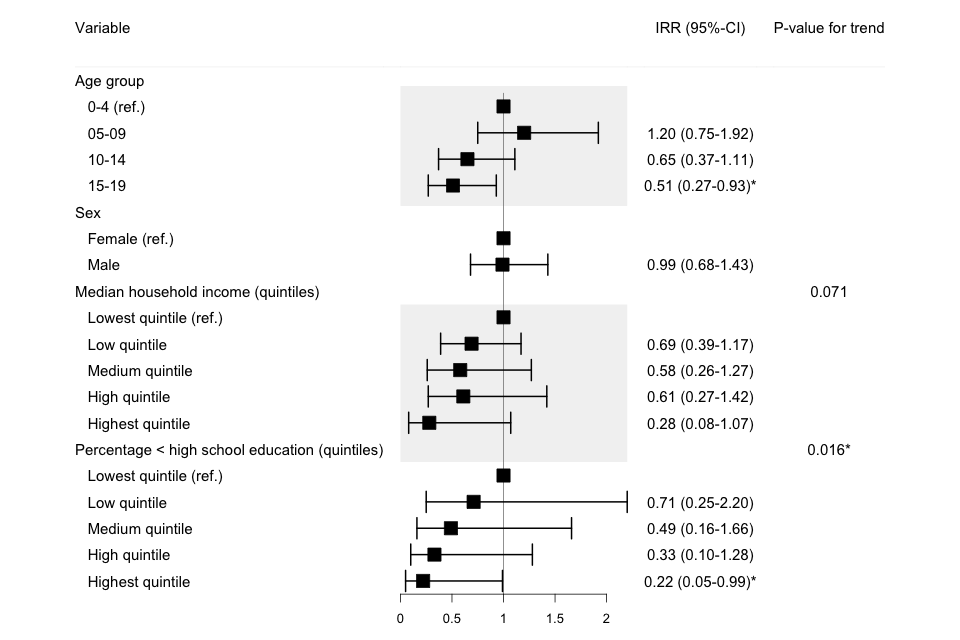


Supplementary figure 2c: Multivariate Poisson regression model of age-adjusted incidence rates (AAIR) among Hispanic medulloblastoma cases.


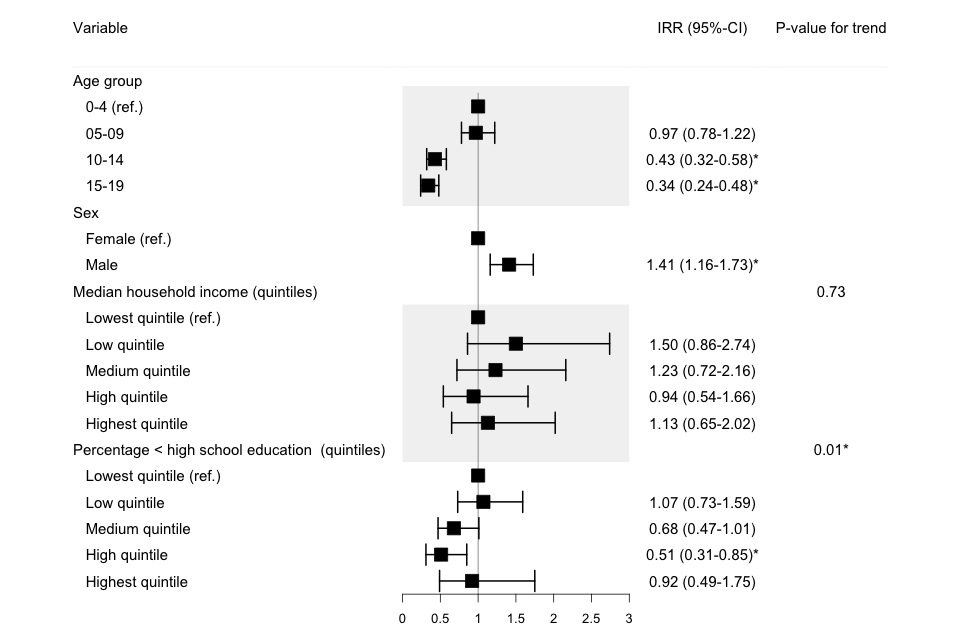


Supplementary figure 2d: Multivariate Poisson regression model of age-adjusted incidence rates (AAIR) among Hispanic medulloblastoma cases with percentage foreign-born.


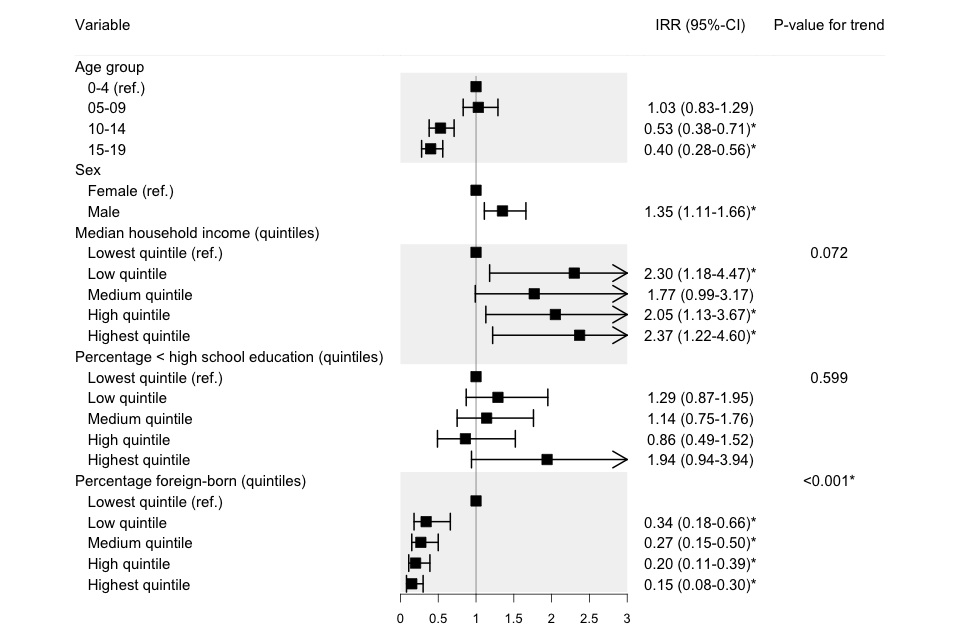


Supplementary figures 2a, 2b, 2c, and 2d legends: Figure 2a, 2b, and 2c are forest plots that depict incidence rate ratios (IRRs) for various risk factors and association with medulloblastoma for non-Hispanic Whites, non-Hispanic Blacks, and Hispanics, respectively. Figure 2d also depicts the IIRs for Hispanics but with percentage foreign-born as an additional variable in the model. IRRs were derived from a Poisson regression model using age-adjusted incidence rates (AAIR) as the dependent variable. Percentage foreign-born was included in the model for Hispanics. Standard population in each category and total standard population were derived from the Census 2000 ACS data.

*Statistically significant incidence rate ratio (IRR).
